# PHLPP2 is regulated by competing endogenous RNA network in pathogenesis of colon cancer

**DOI:** 10.18632/aging.103246

**Published:** 2020-07-07

**Authors:** Hong-Kun Wu, Chang Liu, Xin-Xing Li, Wei Ji, Chen-De Xin, Zhi-Qian Hu, Lin Zhou

**Affiliations:** 1 Department of Laboratory Medicine, Changzheng Hospital, Naval Medical University, Shanghai 200003, P.R. China; 2 Department of General Surgery, Changzheng Hospital, Naval Medical University, Shanghai 200003, P.R. China

**Keywords:** PHLPP2, ceRNA, miRNA, lncRNA, EMT

## Abstract

Recently, homologous pleckstrin-homology (PH)-domain leucine-rich-repeat protein phosphatases (PHLPP2) has been reported as a tumor suppressor in colon cancer. This study aimed to unravel the possible involvement of long noncoding RNAs (lncRNAs) and microRNAs (miRNAs) regulating PHLPP2 in colon cancer. Expressions of candidate lncRNAs and miRNAs were verified by the RT-qPCR and Western blot analyses in colon cancer. The roles of candidate genes in colon cancer were investigated in HT-29 cells *in vitro* and in mouse tumor xenograft model *in vivo*. PHLPP2, a target of miR-141 and miR-424, was downregulated in colon cancer. PHLPP2 upregulation and miR-141 and miR-424 downregulation suppressed the colon cancer cell proliferation, migration, invasion, and epithelial-mesenchymal transition, and promote cell apoptosis, which also resulted in suppression of tumor metastasis and formation. Furthermore, LINC00402, LINC00461, and SFTA1P were identified as the targets of miR-141 and miR-424 and acted as competitive endogenous RNAs (ceRNAs) of PHLPP2. The upregulation of LINC00402, LINC00461, and SFTA1P was verified to enhance the suppressive effects of PHLPP2 in the pathogenesis of colon cancer. Conjointly, our results demonstrated the suppressive effects of PHLPP2 in colon cancer and proved that LINC00402, LINC00461, and SFTA1P acted as ceRNAs of PHLPP2 by competitive binding to miR-141 and miR-424.

## INTRODUCTION

Colon cancer is the third most common malignancy worldwide, accounting for more than 9% of all cancer cases [1, 2]. Despite recent advances in early diagnosis, prevention of tumor progression regarding disease stage (node-positive disease), vascular invasion, obstruction and/or perforation and other clinic pathological implications remained a question for cancer therapeutic [3]. Nevertheless, the quest for the discovery of novel technologies has elucidated the role of specific gene expression and its regulation network associated with colon cancer [4–6]. Peculiarly, the discovery of biological roles of non-coding RNAs (ncRNAs), especially microRNAs (miRNAs) and long noncoding RNAs (lncRNAs) have brought an innovative strategy to unravel the underlying molecular mechanism of tumorigenesis. Moreover, Systematic and integrative analyses have explored the complex regulatory network and interactions of various RNA molecules including competing for endogenous RNAs (ceRNAs) hypothesis [7, 8]. Based on these findings, our study attempted to provide an integrated analysis of network regulation of lncRNAs and miRNAs in colon cancer.

Homologous pleckstrin-homology (PH)-domain leucine-rich-repeat protein phosphatases (PHLPP1-2) has been reported as survival/proliferation suppressors in a variety of human cancers, including colon cancer [9–11]. Moreover, miRNA profiling is considered as a crucial strategy in the diagnosis, prognosis, and treatment of colon cancer [12]. Previously, it has been reported that high-level miR-141 was associated with poor survival in patients with colon cancer, thus identifying, miR-141 as an independent prognostic factor for metastatic colon cancer [13]. Meanwhile, the relationship between the upregulation of the miR-424/503 cluster and tumorigenesis or invasion has also been detected in colon cancer [14]. To date, more than 10,000 lncRNAs have been identified, studying on which provides new opportunities for cancer diagnosis and treatment [15, 16]. Long intergenic non-protein coding RNA 461 (LINC00461) has been reported to be involved in the proliferation, migration, and invasion of glioma cells [17]. Moreover, the Surfactant associated 1, pseudogene (SFTA1P) has been described as a novel diagnostic indicator, in correlation with the survival and progression of lung squamous cell carcinoma [18]. Additionally, another long intergenic non-protein coding i.e., RNA 402 (LINC00402), implicated in metastatic melanoma [19], Thus, based on our previous bioinformatics analysis, these above reported factors were further investigated in our study as a differentially expressed lncRNA.

Hence, Complete genome expression profiles were explored in our work to identify the cancer-specific gene expression and regulatory networks. PHLPP2 was revealed to be downregulated in colon cancer, which was a target of miR-141 and miR-424. Meanwhile, LINC00402 and LINC00461 were found to bind competitively to miR-141 while SFTA1P was determined to bind competitively to miR-424. Therefore, the regulation of the lncRNA ceRNA network was further investigated by proliferation, migration, invasion, and epithelial-mesenchymal transition (EMT) in colon cancer.

## RESULTS

### PHLPP2 Is Identified as the target gene of miR-141 and miR-424

**Figure 1:**
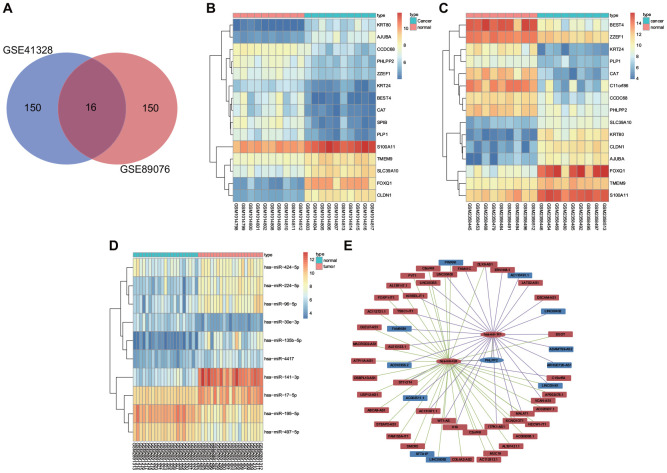
**DEGs and differentially expressed miRNAs are implicated in the occurrence and development of colon cancer.** (**A**) Top 150 DEGs from GSE41328 and GSE89076 by Venn diagrams. (**B**) Differential gene expression heatmap of the GSE41328 dataset. (**C**) Differential gene expression heatmap of the GSE89076 dataset. (**D**) Differential gene expression heatmap of the GSE108153 dataset. (**E**) CeRNA network for identification of ceRNAs associated with colon cancer using the TCGA database as well as miRDB, miRTarBase and TargetScan websites. In Panel (**B**–**D**) the abscissa referred to Sample No., while the ordinate referred to DEGs; the procamp was presented using a histogram in the upper right, where the change in color from top to bottom indicated the expression of the chips from high to low; each rectangle indicated the expression of one gene in one sample, and each column showed the expression of all genes in one sample; the tree diagram was applied to present the results of cluster analysis on the different genes from different samples; the horizontal bar revealed the cancer tissues in red and the adjacent tissues in blue. In Panel (**E**) the upregulated gene was shown in red and the downregulated gene was shown in blue, the lncRNA binding to miR-141 was shown in purple and lncRNA binding to miR-424 was shown in green.

Gene expression information of GSE41328 and GSE89076 in colon cancer were obtained from the GEO database. There were 16 intersection genes from the 2 datasets illustrated by the Venn diagrams as depicted in Figure 1A, with the differential gene expression heatmap of the two datasets shown in Figure 1B, 1C. Our results revealed that KRT80, AJUBA, S100A11, TMEM9, SLC39A10, FOXQ1, and CLDN1 were upregulated whereas the expressions of SPIB, KRT24, PHLPP2, PLP1, BEST4, ZZEF1, CCDC68, CA7, and C11orf86 were downregulated in colon cancer. The KEGG analysis revealed correlations of AJUBA, CLDN1, and PHLPP2 with signaling pathways related to the occurrence and progression of colon cancer (Table 1). The TCGA results indicated that SPIB, KRT24, PHLPP2, PLP1, BEST4, CA7, and C11orf86 were all downregulated, while KRT80, SLC39A10, AJUBA, FOXQ1, and CLDN1 exhibited upregulated levels in colon cancer (Supplementary Figure 1). Besides, miRDB, miRTarBase, and TargetScan websites were applied to identify the miRNA targeting SPIB, KRT24, PHLPP2, PLP1, BEST4, CA7, C11orf86, KRT80, SLC39A10, AJUBA, FOXQ1, and CLDN1. Differentially expressed miRNAs screened from the TCGA database revealed that miR-141, miR-424, miR-17, miR-106a, and miR-32 were highly expressed in colon cancer (Supplementary Figure 2). Furthermore, miR-141, miR-424, and miR-32 were predicted to bind to PHLPP2 mRNA and lead to mRNA degradation. The analysis of the dataset GSE108153 further demonstrated that miR-141 and miR-424 were increased in colon cancer, however, no differential expression of miR-32 was observed in this dataset (Figure 1D). Finally, lncRNAs binding to miR-141 and miR-424 were screened out from the mircode website, with their respective differential expressions verified by the TCGA database. LncRNAs (LINC00402, LINC00461, and SFTA1P) were finally identified as competitive endogenous RNAs (ceRNAs) in association with the occurrence and progression of colon cancer (Figure 1E).

**Table 1:** Primer sequence of related genes for reverse transcription quantitative polymerase chain reaction.

**Gene**	**Primer sequence**
*LINC00402*	F: 5'-TCACTTAACTTCTACGGGTCCAGTT-3'
	R: 5'-GCCATTTGGGGCTGATTAACATTTT-3'
*LINC00461*	F: 5'-GACATTTACGCCACAACCCACG-3'
	R: 5'-AGACAGACCCTCAGATTCCCCA-3'
*SFTA1P*	F: 5'-GAACCGTCTCTCGCGGGACCCTTTA-3'
	R: 5'-CTTTCTACTTACATTCCAAAATAAC-3'
*miR-141*	F: 5'-GGGGTAACACTGTCTGGTAA-3'
	R: 5'-TGCGTGTCGTGGAGTC-3'
*miR-424*	F: 5'-GATCGGATCCGCAGCTCCTGGAAATCAAAT-3'
	R: 5'-GATCGGATCCCCCAGCCTAGCCAGGAATAC-3'
*PHLPP2*	F: 5'-GGACGAGAGGAGGTGGAATA-3'
	R: 5'-CCAGGATCAAGCAAGTTCAGG-3'
*U6*	F: 5'-GCTTCGGCAGCACATATACTAAAAT-3'
	R: 5'-CGCTTCACGAATTTGCGTGTCAT-3'
*GAPDH*	F: 5'-CATCAAGAAGGTGGTGAAGCAG-3'
	R: 5'-AAAGGTGGAGGAGTGGGTGTC-3'

Note: miR-141, microRNA-141; miR-424, microRNA-424; PHPPL2, pleckstrin homology domain leucine-rich repeat protein phosphatase; GAPDH, glyceraldehyde-3-phosphate dehydrogenase.

**Figure 2:**
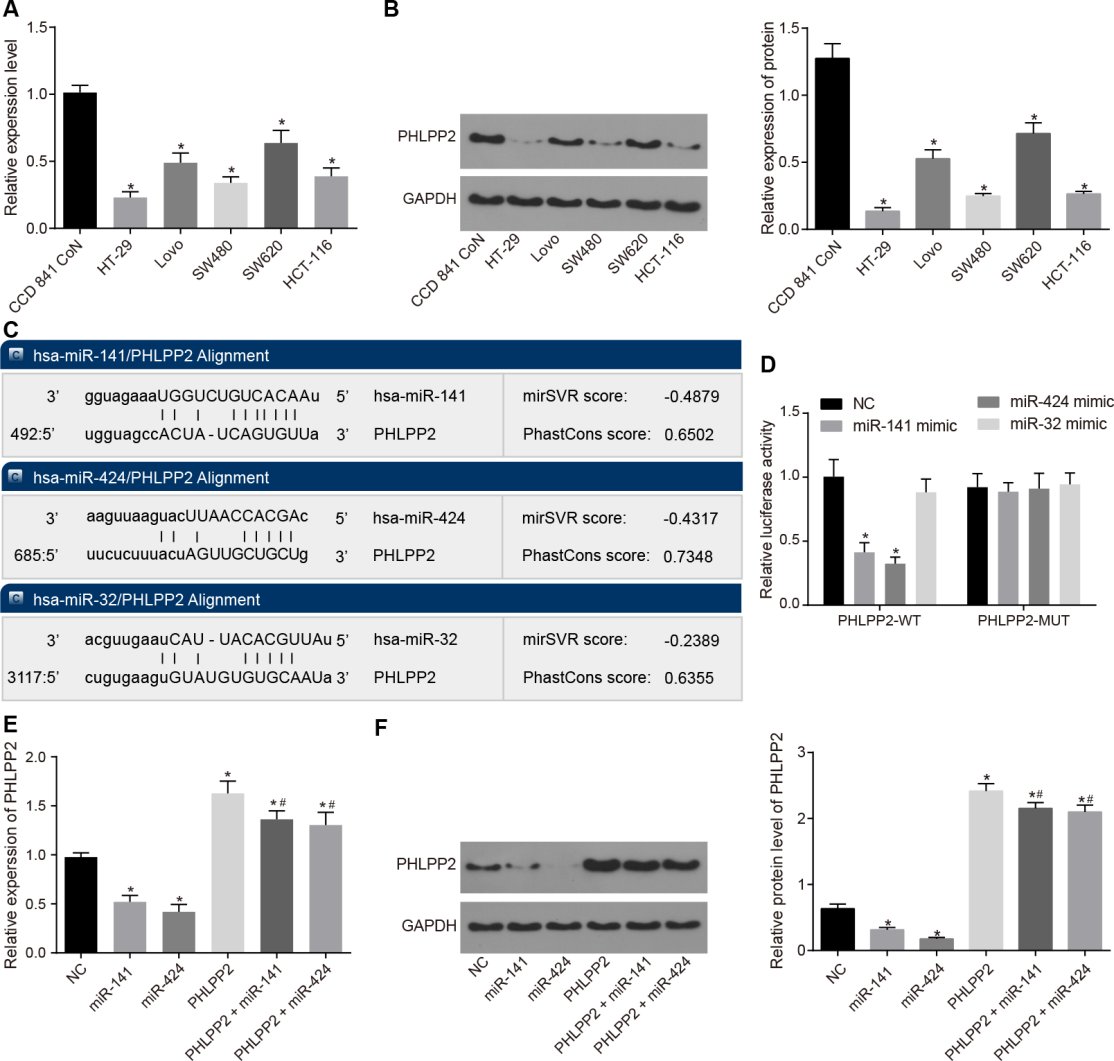
**PHLPP2 is a target gene of miR-141 and miR-424.** (**A**) The determination of PHLPP2 mRNA in CCD 841 CoN, HT-29, SW480, Lovo, HCT-116 and SW620 cell lines by RT-qPCR. (**B**) The expression of PHLPP2 protein in CCD 841 CoN, HT-29, SW480, Lovo, HCT-116, and SW620 cell lines normalized to GAPDH detected by Western blot analysis. (**C**) The sites of miR-141, miR-424 and miR-32 binding to PHLPP2 predicted by the mircoRNA.org website. (**D**) The targeting relation between miR-141, miR-424 or miR-32 and PHLPP2 by the dual-luciferase reporter gene assay. (**E**) The expression of PHLPP2 mRNA influenced by miR-141 and miR-424 in HT-29 cells detected by RT-qPCR. (**F**) The expression of PHLPP2 protein influenced by miR-141 and miR-424 in HT-29 cells normalized to GAPDH detected by Western blot analysis. * *p* < 0.05 *vs.* the NC group; # *p* < 0.05 *vs.* the PHLPP2 group; Measurement data in the present Figure are expressed as the mean ± standard deviation, with comparisons among multiple groups performed using One-Way ANOVA; the experiment was repeated three times independently.

RT-qPCR and Western blot analyses were performed to examine the expression of PHLPP2 in colon epithelial cell line (CCD 841 CoN) and human colon cancer cell lines (HT-29, SW480, Lovo, HCT-116, and SW620), as depicted in Figure 2A, 2B. Our results indicated that compared with the CCD 841 CoN cell line, the human colon cancer cell lines (HT-29, SW480, Lovo, HCT-116 and SW620) presented descended expressions of PHLPP2 mRNA and protein (both *p* < 0.05). Among the five colon cancer cell lines, HT-29 exhibited the lowest PHLPP2 mRNA and protein levels, therefore, the HT-29 cell line was selected as the appropriate cell line for subsequent trials.

Moreover, the TCGA database revealed that miR-141, miR-424, and miR-32 were upregulated in colon cancer (Supplementary Figure 2) whereas the miRDB, miRTarBase, and TargetScan websites indicated that miR-141, miR-424, and miR-32 efficiently bound to PHLPP2 (Figure 2C). These results were further validated by the results of the dual-luciferase reporter gene assay as depicted in Figure 2D. In comparison to the NC group, the luciferase activity was significantly decreased in the miR-141 mimic and miR-424 mimic groups transfected with PHLPP2-WT plasmid (both *p* < 0.05), however, no significant difference was detected among the groups transfected with PHLPP2-MUT plasmid (all *p* > 0.05). No significant difference was detected in miR-32 mimic groups (*p* > 0.05). These results indicated that miR-141 and miR-424 could bind to the PHLPP2 mRNA, however, miR-32 could not. Since reports of miR-32 were controversial in colon cancer [20, 21], further analysis of miR-32 was not included in this study.

To study the regulation of miR-141 and miR-424 on the PHLPP2 gene, the expressions of PHLPP2 mRNA and protein were detected using RT-qPCR and Western blot analyses following the transfection of miR-141 mimic and miR-424 mimic (Figure 2E, 2F). Our results indicated that compared with the NC group, the expressions of PHLPP2 mRNA and protein were significantly increased in the PHLPP2, PHLPP2 + miR-141 and PHLPP2 + miR-424 groups whereas the decreased levels of these factors were observed in the miR-141 and miR-424 groups (all *p* < 0.05). When compared with the PHLPP2 group, the PHLPP2 + miR-141 and PHLPP2 + miR-424 groups exhibited significant downregulation in PHLPP2 mRNA and its protein expressions (all *p* < 0.05). Therefore, miR-141 and miR-424 were confirmed to inhibit the transcription and translation of PHLPP2.

### Expression of PHLPP2 decreases while miR-141 and miR-424 increases in colon cancer

**Figure 3:**
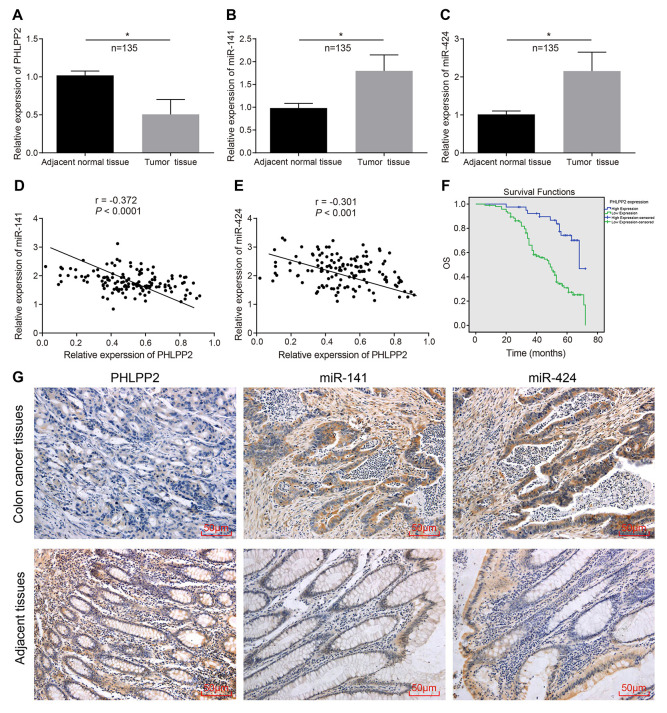
**The expression of PHLPP2 decreases while that of miR-141 and miR-424 increases in colon cancer tissues.** (**A**) Measurement of the expression of PHLPP2 mRNA in colon cancer tissues and adjacent tissues by RT-qPCR. (**B**) Determination of the expression of miR-141 in colon cancer tissues and adjacent tissues by RT-qPCR. (**C**) Determination of the expression of miR-424 in colon cancer tissues and adjacent tissues by RT-qPCR. (**D**) Pearson’s Correlation analysis of PHLPP2 and miR-141 in colon cancer. (**E**) Pearson’s Correlation analysis of PHLPP2 and miR-424 in colon cancer. (**F**) Survival time of patients with high and low expression of PHLPP2 analyzed by Kaplan-Meier survival analysis. (**G**) Immunohistochemistry and ISH were applied to identify the expression of PHLPP2, miR-141, and miR-424 in colon cancer tissues and adjacent tissues (200 ×). * *p* < 0.05 *vs.* the adjacent tissues; Measurement data in this Figure are expressed as the mean ± standard deviation, with the comparisons between the two groups conducted using paired *t*-test, n = 135; the experiment was repeated three times independently.

RT-qPCR was applied to determine the expression of PHLPP2 mRNA, miR-141, and miR-424 in both the colon cancer and adjacent tissues. In comparison to the adjacent tissues, expression of PHLPP2 was significantly decreased while miR-141 and miR-424 exhibited increased expression in colon cancer tissues (Figure 3A–3C). Pearson's Correlation Analysis indicated negative correlations between PHLPP2 and miR-141 or miR-424, respectively (all *p* < 0.05) (Figure 3D, 3E). The ISH also showed an increase in the expression of miR-141 and miR-424 in colon cancer in comparison to the adjacent tissues (Figure 3G).

Immunohistochemistry methods were applied to explore the expression of PHLPP2 in the colon cancer tissues and adjacent tissues (Figure 3G). The PHLPP2 was found to be localized in the nucleus and cytoplasm of cells whereas the expression of PHLPP2 in the colon cancer tissues was notably lowered than that in adjacent tissues. To further evaluate the relationship between the expression of PHLPP2 and the prognosis of patients with colon cancer, the expression of PHLPP2 in colon cancer tissues was analyzed in patients with colon cancer. The survival time of patients with different PHLPP2 expression levels was analyzed by the Kaplan-Meier survival curves and our result indicated the reduction in overall survival time of colon cancer patients with low expressions of PHLPP2 compared to patients with high expression of PHLPP2 (*p* < 0.05) (Figure 3F).

### Overexpressed PHLPP2 inhibits proliferation, migration, invasion, and EMT in HT-29 cells

**Figure 4:**
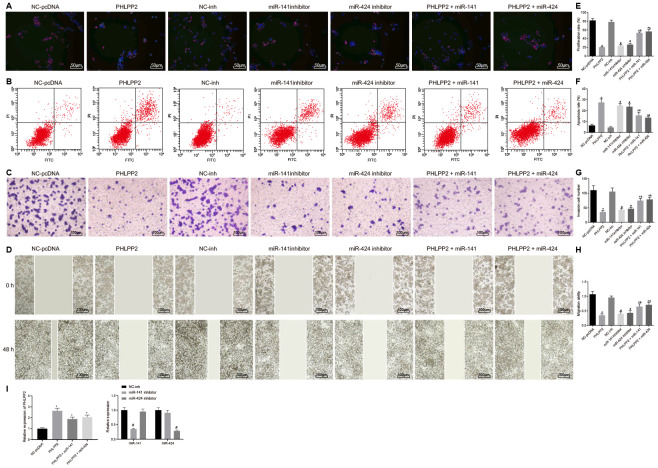
**Overexpressed PHLPP2 inhibits the proliferation, migration, invasion of HT-29 cells.** HT-29 cells were transfected with NC-pcDNA, PHLPP2, NC-inh, miR-141 inhibitor, miR-424 inhibitor, PHLPP2 + miR-141 or PHLPP2 + miR-424. (**A**, **E**) EdU labeling detected HT-29 cell proliferation (200 ×). (**B**, **F**) Flow cytometry analyzed apoptotic HT-29 cells. (**C**, **G**) Transwell assay detected HT-29 cell migration (100 ×). (**D**, **H**) Scratch test detected invasive HT-29 cells 48 h after treatment. (**I**) Transfection efficiency detected by RT-qPCR. * *p* < 0.05 *vs.* the NC-pcDNA group; # *p* < 0.05 *vs.* the NC-inh group; &, *p* < 0.05 *vs.* the PHLPP2 group; Measurement data in this Figure are expressed as the mean ± standard deviation, while comparisons among multiple groups were conducted using One-Way ANOVA; the experiment was repeated three times independently.

To further elucidate the biological function of PHLPP2, the proliferation, migration, invasion, and apoptosis of HT-29 cell line were assessed in the presence of overexpression of PHLPP2, or interference of miR-141 and miR-424 (Figure 4A–4H). RT-qPCR was firstly performed for the detection of transfection efficiency (Figure 4I). The PHLPP2 expression was found to significantly increase in the PHLPP2, PHLPP2 + miR-141 and PHLPP2 + miR-424 groups in comparison to the NC-pcDNA group, while a highest PHLPP2 expression was found in the PHLPP2 group. Relative to the NC-inh group, the miR-141 expression was significantly decreased in the miR-141 inhibitor group whereas miR-424 expression did not differ significantly, similarly, no significant difference was witnessed regarding miR-141 expression in the miR-424 inhibitor group whereas miR-424 was significantly downregulated. Compared with the NC-pcDNA group, the proliferation, migration, and invasion of HT-29 cell line were suppressed in the PHLPP2, PHLPP2 + miR-141 and PHLPP2 + miR-424 groups, respectively while the rate of apoptosis was elevated (all *p* < 0.05). In comparison to the NC-inh group, cell proliferation, migration, and invasion were decreased, whereas apoptosis was increased in the miR-141 inhibitor and miR-424 inhibitor groups (all *p* < 0.05). Additionally, the PHLPP2 + miR-141 and PHLPP2 + miR-424 groups exhibited higher cell proliferation, migration, and invasion whilst lower apoptosis rate than the PHLPP2 group (all *p* < 0.05). Thus, it indicates that PHLPP2 exerted an inhibitory role in cell proliferation, migration, and invasion, as well as promoted the cell apoptosis.

Since the PHLPP2 expression was highest in the SW620 colon cancer cell line among the colon cancer cell lines, PHLPP2 was then upregulated in the SW620 cell line to further investigate the biological function of PHLPP2 in colon cancer cells followed by cellular behavior evaluation. RT-qPCR for detection of transfection efficiency revealed that PHLPP2 expression was significantly upregulated in the PHLPP2 group compared with the NC-pcDNA group (Supplementary Figure 3A), while cell proliferation, migration, and invasion were suppressed and apoptosis was promoted in the PHLPP2 group (all *p* < 0.05) (Supplementary Figure 3B–3E). Taken together, these findings demonstrated the regulatory role of PHLPP2 in cellular behaviors of colon cancer cells.

**Figure 5:**
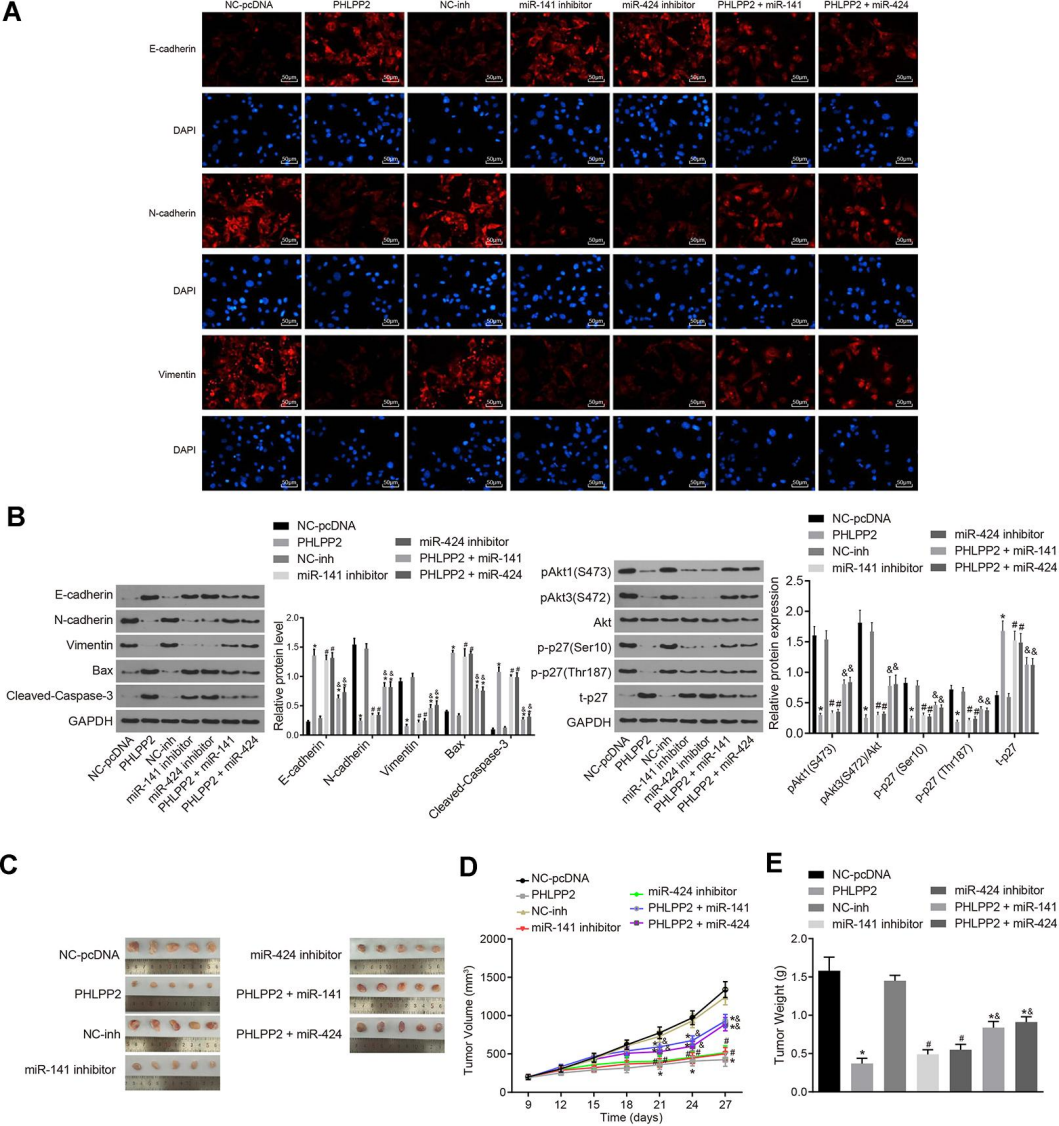
**Overexpressed PHLPP2 suppresses the EMT process and tumorigenicity of HT-29 cells.** HT-29 cells were transfected with NC-pcDNA, PHLPP2, NC-inh, miR-141 inhibitor, miR-424 inhibitor, PHLPP2 + miR-141 or PHLPP2 + miR-424. (**A**) The expressions of EMT-related factors (E-cadherin, N-cadherin, and Vimentin) detected by immunofluorescence assay in HT-29 cells (200 ×). (**B**) Western blot analysis of EMT-related factors (E-cadherin, N-cadherin and Vimentin), apoptosis factors (Bax and cleaved Caspase-3) and Akt3-p27 pathway-related proteins (Akt1, Akt3, and p27) in HT-29 cells normalized to GAPDH. (**C**) The representative images of formed tumors in nude mice. (**D**) Tumor growth of nude mice. (**E**) Tumor weight of nude mice. * *p* < 0.05 *vs.* the NC-pcDNA group; # *p* < 0.05 *vs.* the NC-inh group; & *p* < 0.05 *vs.* the PHLPP2 group; Measurement data in this Figure expressed as the mean ± standard deviation, with comparisons between multiple groups conducted using One-Way ANOVA (n = 5); the experiment was repeated three times independently.

To elucidate the mechanism of the PHLPP2 effect on cell migration and invasion, analyses of EMT in HT-29 cells were performed after PHLPP2 transfection. The expression of E-cadherin, Bax, and cleaved Caspase-3 were increased in the PHLPP2, PHLPP2 + miR-141 and PHLPP2 + miR-424 groups compared with the NC-pcDNA group whereas N-cadherin and Vimentin were decreased (all *p* < 0.05) as shown in Figure 5A, 5B. In comparison to the NC-inh group, the expressions of E-cadherin, Bax, and cleaved Caspase-3 were elevated and the expressions of N-cadherin and Vimentin were declined in the miR-141 inhibitor and miR-424 inhibitor groups (all *p* < 0.05). The PHLPP2 + miR-141 and PHLPP2 + miR-424 groups exhibited a lower level of E-cadherin, Bax, and cleaved Caspase-3, and higher level of N-cadherin and Vimentin than that in the PHLPP2 group (all *p* < 0.05).

Furthermore, the effects of miR-141 and miR-424 on PHLPP2-Akt3-p27 pathway were investigated by Western blot (Figure 5B). After the transfection of PHLPP2, the levels of p-Akt3 (S472) and p-Akt1 (S473) were significantly decreased in HT-29 cells combined with increased t-P27. Additionally, the level of p-p27 (Ser10) and p-p27 (Thr187) phosphorylation were found to be decreased in the HT-29 cells after PHLPP2 transfection (all *p* < 0.05). Our results confirmed that PHLPP2 could dephosphorylate Akt and oppose the action of Akt3 on phosphorylation of p27. Meanwhile, similar results were also detected in the miR-141/miR-424 inhibitor groups (all *p* < 0.05). The PHLPP2 + miR-141 and PHLPP2 + miR-424 groups exhibited increased level of p-Akt3 (S472), p-p27(Ser10), and p-p27 (Thr187) (all *p* < 0.05). These aforementioned results revealed that the overexpression of PHLPP2, as well as the inhibition of miR-141 or miR-424, could inhibit the proliferation, migration, invasion, and EMT of HT-29 cells. The overexpression of miR-141 or miR-424 could suppress the inhibition effect of PHLPP2 on development of colon cancer.

In an attempt to validate the inhibitory role of PHLPP2 in colon cancer, tumor xenografts in nude mice were applied to evaluate the effect of PHLPP2 on tumor growth. As depicted in the results (Figure 5C–5E), tumor weight and volume in nude mice were reduced, moreover, the tumor growth was also inhibited in the PHLPP2, PHLPP2 + miR-141 and PHLPP2 + miR-424 groups compared with the NC-pcDNA group (all *p* < 0.05). Similar results were detected in the miR-141 and miR-424 inhibitor groups. Furthermore, the nude mice in the PHLPP2 + miR-141 and PHLPP2 + miR-424 groups indicated the increased tumor weight and volume as well as a faster rate of tumor growth when compared to the PHLPP2 group (all *p* < 0.05). Based on this aforementioned data, as well as the results of the *in vivo* and *in vitro* experiments, the overexpression of PHLPP2 and inhibition of miR-141 or miR-424 were determined to inhibit the tumor formation whereas the overexpression of miR-141 or miR-424 could suppress the inhibitory role of PHLPP2 in relation to the development of colon cancer.

### LINC00402, LINC00461 and SFTA1P Act as Targets of miR-141 or miR-424 and lead to enhanced expression of PHLPP2

**Figure 6:**
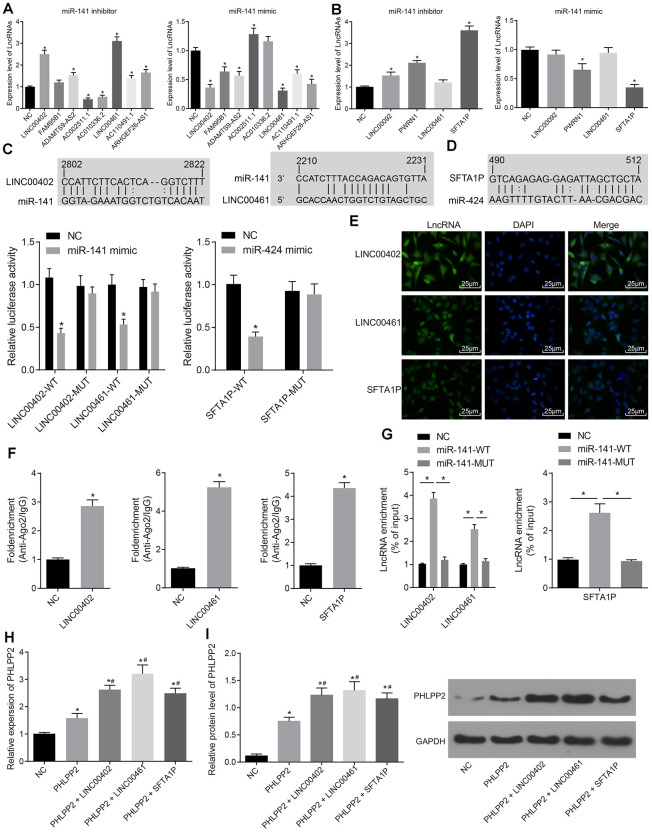
**The expression of PHLPP2 is regulated by LINC00402 and LINC00461 competitively binding to miR-141 as well as SFTA1P competitively binding to miR-424.** (**A**) RT-qPCR was conducted to measure the expressions of lncRNAs when HT-29 cells were transfected with miR-141 inhibitor or mimic. (**B**) RT-qPCR was performed to measure the expressions of lncRNAs when the HT-29 cells were transfected with miR-424 inhibitor or mimic. (**C**) Dual-luciferase reporter gene assay verified LINC00402 and LINC00461 binding to miR-141. (**D**) Dual-luciferase reporter gene assay verified SFTA1P binding to miR-424. (**E**) FISH technology was utilized to identify the subcellular localization of LINC00402, LINC00461, and SFTA1P in the colon cancer (400 ×). (**F**) RNA pull-down was performed to show the relationship of LINC00402 and LINC00461 binding to miR-141, SFTA1P binding to miR-424. (**G**) LINC00402, LINC00461 and SFTA1P binding to Ago2 as reflected by the RIP assay. (**H**) Relative expression of PHLPP2 mRNA determined by RT-qPCR. (**I**) Protein expression of PHLPP2 protein normalized to GAPDH determined by Western blot analysis. * *p* < 0.05 *vs.* the NC group; # *p* < 0.05 *vs.* the PHLPP2 group; Measurement data in this Figure expressed as the mean ± standard deviation, while comparisons among multiple groups were conducted using One-Way ANOVA; the experiment was repeated three times independently.

To further explore the regulation of PHLPP2 in colon cancer, more targets of miR-141 and miR-424 were analyzed. According to the TCGA and miRcode database, eight miR-141 target lncRNAs and four miR-424 target lncRNAs were identified. Expressions of these lncRNAs were analyzed in HT-29 cells transfected with miR-141 or miR-424 (Figure 6A, 6B). Our results indicated that the expression of LINC00402, ADAMTS9-AS2, LINC00461, AC110491.1, and ARHGEF26-AS1 were significantly increased after downregulation of miR-141 and vice versa (all *p* < 0.05). Among these lncRNAs, LINC00402 and LINC00461, with the most significantly increased level, were selected for further analysis. Meanwhile, expressions of LINC00092, PWRN1, and SFTA1P were also increased in the HT-29 cells transfected with miR-424 inhibitor (all *p* < 0.05); whereas the levels of PWRN1 and SFTA1P were decreased in HT-29 cells in response to the overexpression of miR-424 (all *p* < 0.05). SFTA1P was selected for subsequent analysis. The FISH analysis revealed that LINC00402 was localized in the cytoplasm while LINC00461 and SFTA1P were located in cytoplasm and nucleus, respectively (Figure 6E).

The above-described results were further verified by the dual-luciferase reporter gene assay (Figure 6C, 6D). The luciferase activity was significantly decreased in the LINC00402-WT and LINC00461-WT groups transfected with miR-141 mimic (both *p* < 0.05). While no significant difference was detected in the LINC00402-MUT or LINC00461-MUT groups (*p* > 0.05). The luciferase activity in the cells treated with SFTA1P-WT was also decreased after miR-424 mimic transfection (*p* < 0.05). However, no significant difference was detected in SFTA1P-MUT (*p* > 0.05). Our results indicated that miR-141 could specifically bind to the LINC00402 and LINC00461 as well as miR-424 to SFTA1P, which conversely indicated that LINC00402, LINC00461, and SFTA1P could act as ceRNAs to regulate the expression of PHLPP2 by competitive binding to miR-141 or miR-424.

The combination of three lncRNAs i.e, LINC00402, LINC00461, and SFTA1P, with Ago2, was evaluated by RIP assay whereas the combination of lncRNAs (LINC00402, LINC00461, and SFTA1P) with miRNAs (miR-141 or miR-424) was also assessed by RNA pull-down experiments. The results of the RIP assay revealed that the combination of LINC00402, LINC00461 or SFTA1P to Ago2 were significantly upregulated compared with control (both *p* < 0.05), confirming that all these lncRNAs could bind to Ago2 (Figure 6F). The combination of LINC00402 and LINC00461 with miR-141-WT was upregulated compared with the NC and miR-141-MUT groups (both *p* < 0.05) while similar results were detected between SFTA1P and miR-424-WT (*p* < 0.05) (Figure 6G). These results collectively suggest that three lncRNAs including LINC00402, LINC00461, and SFTA1P could bind to both Ago2 and miR-141/miR-424. Moreover, their interaction with Ago2 might influence its association with miR-141/miR-424.

To further clarify the relationship between lncRNAs (LINC00402, LINC00461, and SFTA1P) and PHLPP2, expressions of PHLPP2 in cells transfected with LINC00402, LINC00461 or SFTA1P, RT-qPCR and Western blot analyses were performed. The PHLPP2, PHLPP2 + LINC00402, PHLPP2 + LINC00461, and PHLPP2 + SFTA1P groups exhibited the higher level of PHLPP2 compared with the NC group (all *p* < 0.05) (Figure 6H). The expressions of PHLPP2 mRNA and protein were elevated in the PHLPP2 + LINC00402, PHLPP2 + LINC00461, and PHLPP2 + SFTA1P groups compared with the PHLPP2 group (all *p* < 0.05) (Figure 6I). These results confirmed that LINC00402, LINC00461, and SFTA1P possess the ability to enhance PHLPP2 expression.

### Overexpression of LINC00402, LINC00402, and SFTA1P inhibits miR-141 or miR-424 regulation of PHLPP2 and suppresses aggressive phenotype in HT-29 cells

**Figure 7:**
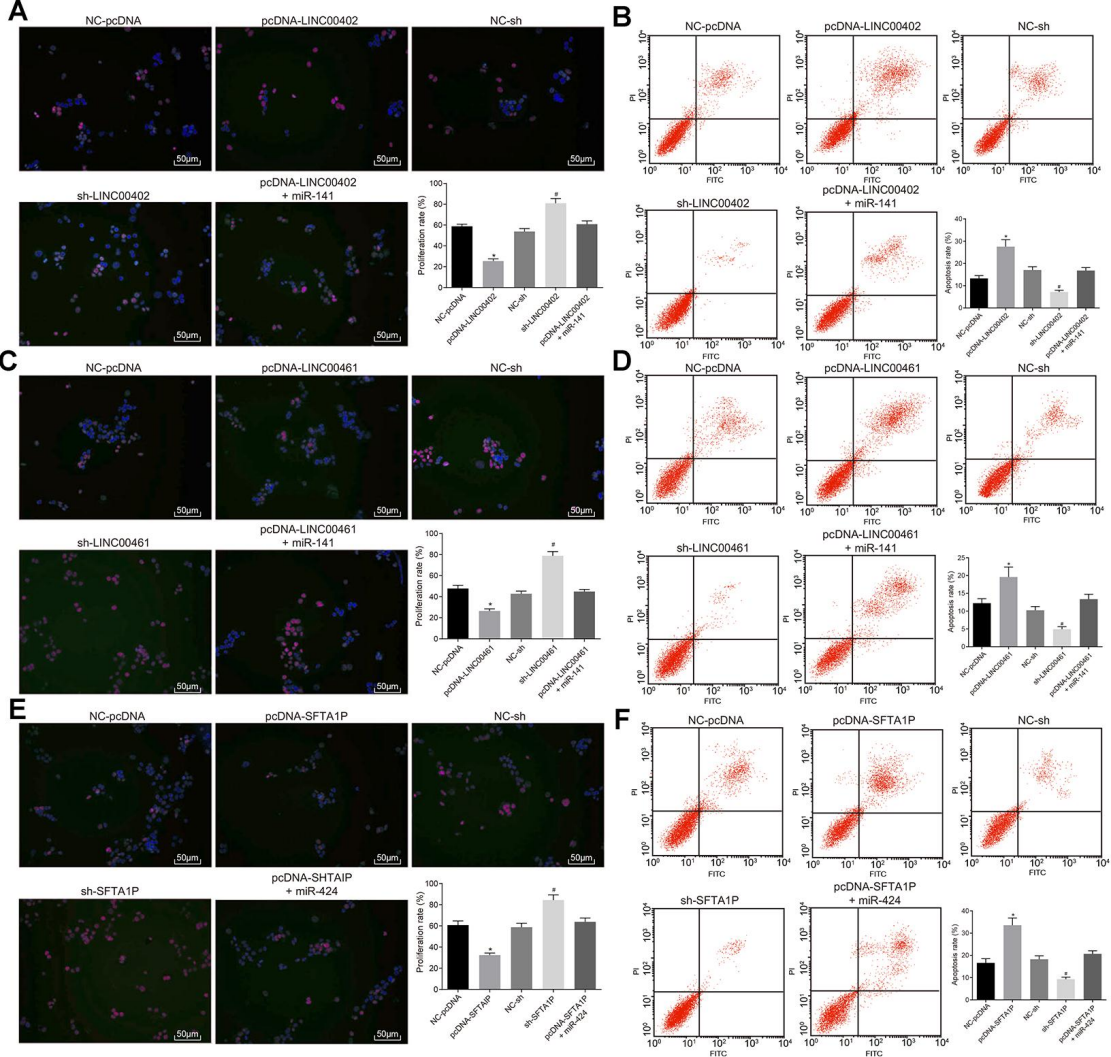
**Overexpressed LINC00402, LINC00461, and SFTA1P inhibit the proliferation and promote apoptosis of HT-29 cells. (A**) Fluorescence image by EdU labeling (200 ×) evaluated cell proliferation when HT-29 cells were transfected with overexpressed LINC00402. (**B**) Fluorescence image by flow cytometry (200 ×) measured cell apoptosis when HT-29 cells were transfected with overexpressed LINC00402. (**C**) Fluorescence image by EdU labeling (200 ×) evaluated cell proliferation when HT-29 cells were transfected with overexpressed LINC00461. (**D**) Fluorescence image by flow cytometry (200 ×) assessed cell apoptosis when HT-29 cells were transfected with overexpressed LINC00461. (**E**) Fluorescence image by EdU labeling (200 ×) cell proliferation evaluated when HT-29 cells were transfected with overexpressed SFTA1P. (**F**) Fluorescence image by flow cytometry (200 ×) assessed cell apoptosis when HT-29 cells were transfected with overexpressed SFTA1P. * *p* < 0.05 *vs.* the NC-pcDNA group; # *p* < 0.05 *vs.* the NC-sh group; & *p* < 0.05 *vs.* the PHLPP2 group. Measurement data (proliferation rate and apoptosis rate) in this Figure were expressed as the mean ± standard deviation, and comparisons among multiple groups were conducted using One-Way ANOVA; the experiment was repeated three times independently.

**Figure 8:**
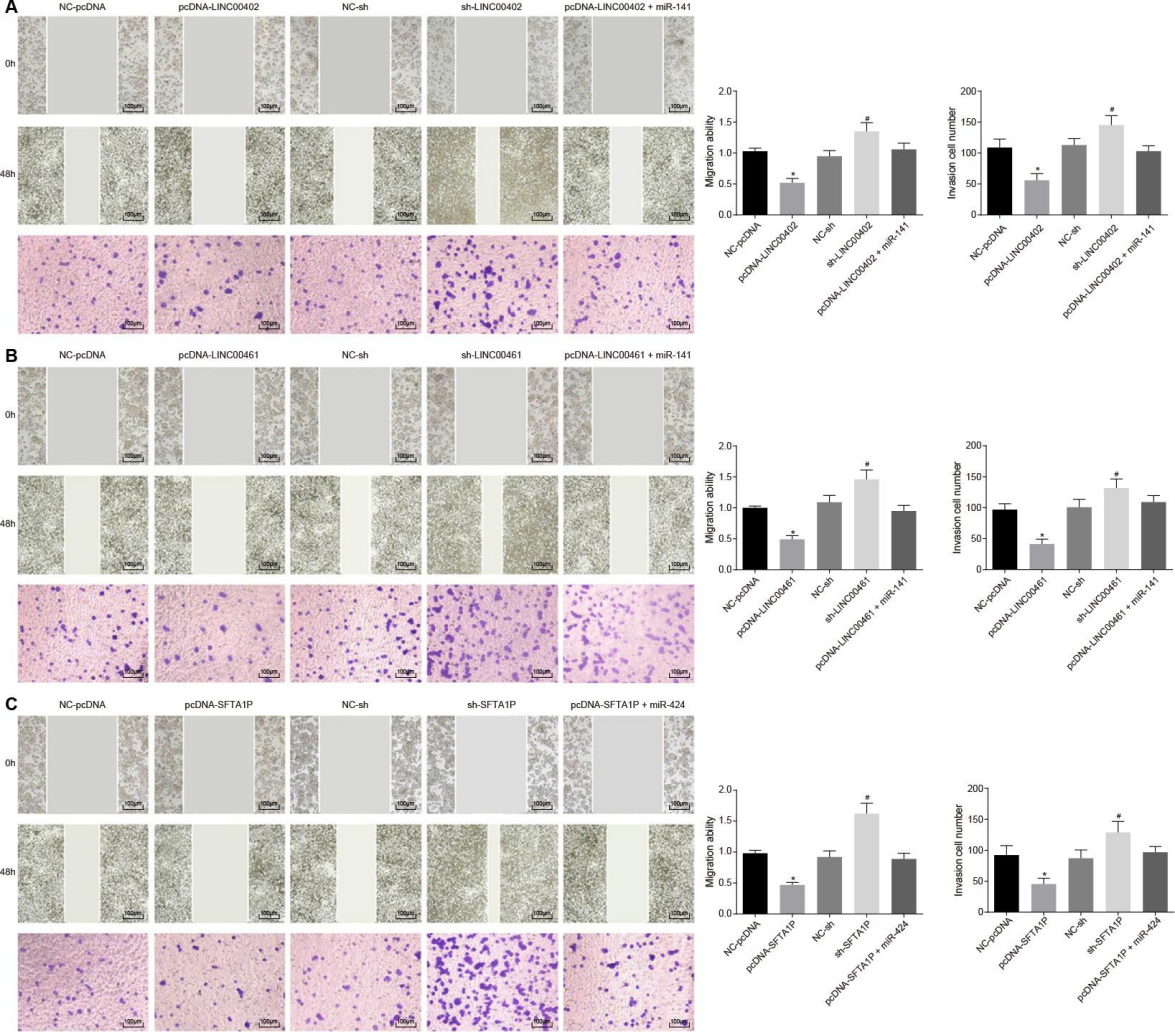
**Overexpressed LINC00402, LINC00461, and SFTA1P inhibit the migration and invasion of HT-29 cells.** (**A**) Scratch test and Transwell assay (100 ×) detected cell migration and invasion in HT-29 cells transfected with overexpressed LINC00402. (**B**) Scratch test and Transwell assay (100 ×) assessed cell migration and invasion when HT-29 cells were transfected with overexpressed LINC00461. (**C**) Scratch test and Transwell assay (100 ×) assessed cell migration and invasion when HT-29 cells were transfected with overexpressed SFTA1P. * *p* < 0.05 *vs.* the NC-pcDNA group; # *p* < 0.05 *vs.* the NC-sh group; & *p* < 0.05 *vs.* the PHLPP2 group. Measurement data in this Figure were expressed as the mean ± standard deviation, and comparisons among multiple groups were conducted using One-Way ANOVA; the experiment was repeated three times independently.

To evaluate the effects of three above described lncRNAs on the aggressive phenotype of HT-29 cells these lncRNAs were transfected into HT-29 cells, respectively. As shown in Figure 7A, 7B and Figure 8A, compared with the NC-pcDNA group, the proliferation, migration, and invasion of HT-29 cells were all reduced in the pcDNA-LINC00402 group, while the apoptosis was enhanced (all *p* < 0.05). No significant differences were detected between the NC-pcDNA and pcDNA-LINC00402 + miR-141 groups (all *p* > 0.05). Compared to the NC-sh group, the sh-LINC00402 group exhibited increased proliferation, migration, and invasion, however, decreased cell apoptosis (all *p* < 0.05) was observed. Similar results were detected in LINC00461 (Figure 7C, 7D and Figure 8B) and SFTA1P (Figure 7E, 7F and Figure 8C). These results indicated that the overexpression of LINC00402, LINC00461 or SFTA1P could suppress cell proliferation, migration, and invasion while promoting cell apoptosis in colon cancer, moreover, miR-141/miR-424 could attenuate the inhibition effect of these three targets.

**Figure 9:**
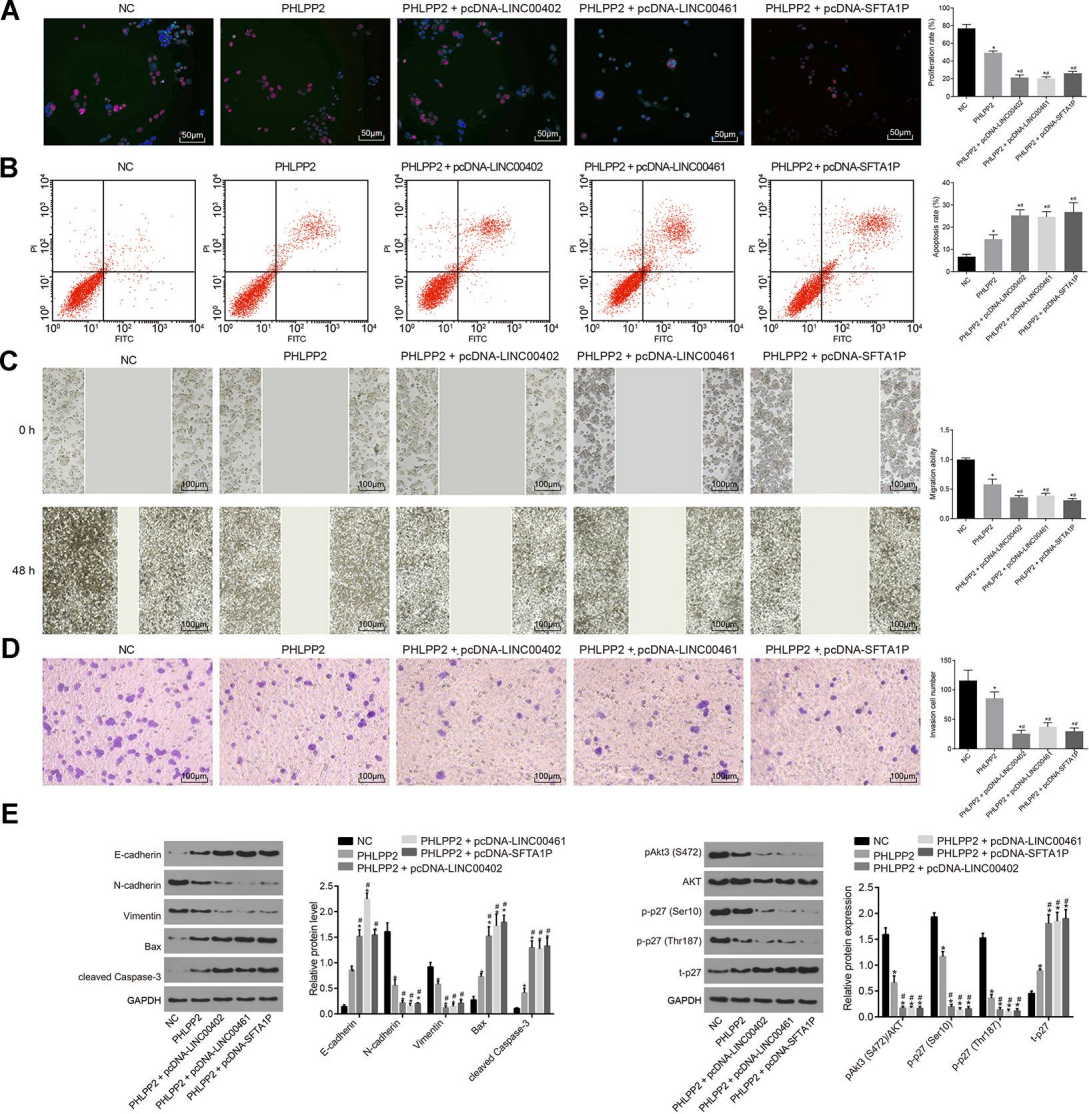
**Overexpression of LINC00402, LINC00461, and SFTA1P enhances the inhibition of PHLPP2 on HT-29 cell proliferation, migration and invasion, and promotion on cell apoptosis.** HT-29 cells were transfected with NC, PHLPP2, PHLPP + pcDNA-LINC00402, PHLPP + pcDNA-LINC00461 or PHLPP + pcDNA-SFTA1P. (**A**) Fluorescence image (200 ×) by EdU labeling showed cell proliferation. (**B**) Flow cytometry detected cell apoptosis. (**C**) Scratch test (100 ×) revealed cell migration. (**D**) Transwell assay (100 ×) suggested cell invasion. (**E**) Western blot analysis of EMT-related proteins (E-cadherin, N-cadherin, and Vimentin), apoptosis-related proteins (cleaved Caspase-3 and Bax) and Akt3-p27 pathway-related proteins (Akt1, Akt3, and p27) in HT-29 cells normalized to GAPDH. * *p* < 0.05 *vs.* the NC group; # *p* < 0.05 *vs.* the PHLPP2 group. Measurement data in this Figure were expressed as the mean ± standard deviation, with comparisons among multiple groups conducted by One-Way ANOVA; the experiment was repeated three times independently.

To evaluate the interaction between three lncRNAs and PHLPP2 *in vitro*, HT-29 cells were co-transfected with PHLPP2 and LINC00402, LINC00402 or SFTA1P (Figure 9A–9D). Our results demonstrated that proliferation, migration, and invasion of HT-29 cells were decreased while apoptosis was increased in the PHLPP2, PHLPP2 + pcDNA-LINC00402, PHLPP2 + pcDNA-LINC00461, and PHLPP2 + pcDNA-SFTA1P groups (all *p* < 0.05). Meanwhile, the PHLPP2 + pcDNA-LINC00402, PHLPP2 + pcDNA-LINC00461, and PHLPP2 + pcDNA-SFTA1P groups exhibited the reduced proliferation, migration, and invasion and enhanced apoptosis compared with the PHLPP2 group (all *p* < 0.05). These results confirmed that overexpression of LINC00402, LINC00402, and SFTA1P could enhance the suppressive effect of PHLPP2 in colon cancer cells.

Further experiments showed that EMT-related factor E-cadherin and apoptosis factors cleaved Caspase-3 and Bax were all upregulated in the PHLPP2, PHLPP2 + pcDNA-LINC00402, PHLPP2 + pcDNA-LINC00461, and PHLPP2 + pcDNA-SFTA1P groups while N-cadherin and Vimentin were downregulated (Figure 9E) (all *p* < 0.05). Compared with the PHLPP2 group, expression of E-cadherin, cleaved Caspase-3, and Bax was upregulated whereas N-cadherin and Vimentin were decreased in the PHLPP2 + pcDNA-LINC00402, PHLPP2 + pcDNA-LINC00461 and PHLPP2 + pcDNA-SFTA1P groups (all *p* < 0.05). Furthermore, the effects of three lncRNAs on the PHLPP2-Akt3-p27 pathway were also explored (Figure 9E). After transfection with PHLPP2, all three lncRNAs significantly inhibited levels of p-Akt3 (S472), p-p27 (Ser10), and p-p27 (Thr187), combined with a significant increase of t-P27 (all *p* < 0.05). The results showed that overexpression of LINC00402, LINC00402, and SFTA1P enhanced the suppression of PHLPP2 on EMT in colon cancer cells.

### Overexpression of LINC00402, LINC00461, and SFTA1P inhibits miR-141 Or miR-424 regulation of PHLPP2 and suppresses tumor formation and metastasis in mouse tumor xenograft model

**Figure 10:**
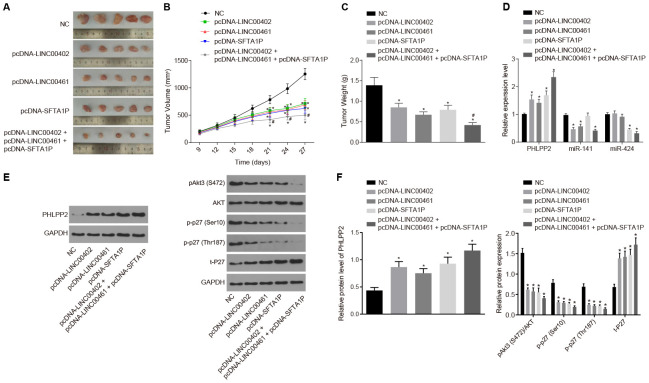
**The overexpression of LINC00402, LINC00461, and SFTA1P suppresses tumor formation in nude mice.** Nude mice were injected with cell suspension carrying HT-29 cells transfected with NC, PHLPP2, PHLPP + pcDNA-LINC00402, PHLPP + pcDNA-LINC00461 or PHLPP + pcDNA-SFTA1P. (**A**) The representative images of formed tumors among the nude mice. (**B**) Tumor growth curve of nude mice. (**C**) Tumor weight of nude mice. (**D**) RT-qPCR was carried out to detect the expressions of PHLPP2 mRNA, miR-141 and miR-424 in nude mice (**E**, **F**) Protein levels of PHLPP2 proteins, Akt3 and p27 in nude mice normalized to GAPDH detected by Western blot analysis. * *p* < 0.05 *vs.* the NC group; # *p* < 0.05 *vs.* the pcDNA-LINC00402, pcDNA-LINC00461 and pcDNA-SFTA1P groups. Measurement data in this Figure were expressed as the mean ± standard deviation, and comparisons among multiple groups conducted using One-Way ANOVA (n = 5); the experiment was repeated three times independently.

Tumor xenograft mice were constructed to investigate the role of lncRNAs in colon cancer *in vivo*. As illustrated in Figure 10A–10C, the growth of tumor was inhibited in the pcDNA-LINC00402, pcDNA-LINC00461, pcDNA-SFTA1P, and pcDNA-LINC00402 + pcDNA-LINC00461 + pcDNA-SFTA1P groups (all *p* < 0.05), among which the pcDNA-LINC00402 + pcDNA-LINC00461 + pcDNA-SFTA1P groups showed the most significant suppression (all *p* < 0.05). These results indicated the existence of a synergistic effect on tumor suppression between LINC00402 and SFTA1P, as well as LINC00461 and SFTA1P, which contributes to the suppression of tumor formation.

RT-qPCR and Western blot assay were employed to examine the ceRNA network interaction between PHLPP2, 3 lncRNAs, and 2 miRNAs *in vivo* (Figure 10D–10F). In contrast to the NC group, the expression of PHLPP2 was downregulated by miR-141 (all *p* < 0.05), which was upregulated by three lncRNAs (all *p* < 0.05). Similar results were detected in miR-424. These results supported that LINC00402, LINC00461, and SFTA1P could act as ceRNAs and de-repress the inhibition of miR-141 or miR-424 on PHLPP2.

Furthermore, the levels of Akt3 and p27, in xenograft tumor excised from nude mice, were explored by Western blot analysis (Figure 10E, 10F). All the pcDNA-LINC00402, pcDNA-LINC00461, pcDNA-SFTA1P, and pcDNA-LINC00402 + pcDNA-LINC00461 + pcDNA-SFTA1P groups had significantly inhibited the levels of p-Akt3, p-p27(Ser10) and p-p27(Thr187), combined with increased t-P27 expression (all *p* < 0.05).

The aforementioned results indicated that both LINC00402 and LINC00461 inhibited the expression of miR-141, as well as SFTA1P, inhibited miR-424. Meanwhile, all three lncRNAs were found to enhance the level of PHLPP2 resulting in inactivation of the Akt3-p27 pathway. Notably, the combined effects of three lncRNAs could synthetically enhance the upregulation of PHLPP2 and downregulation of miR-141 or miR-424.

**Figure 11:**
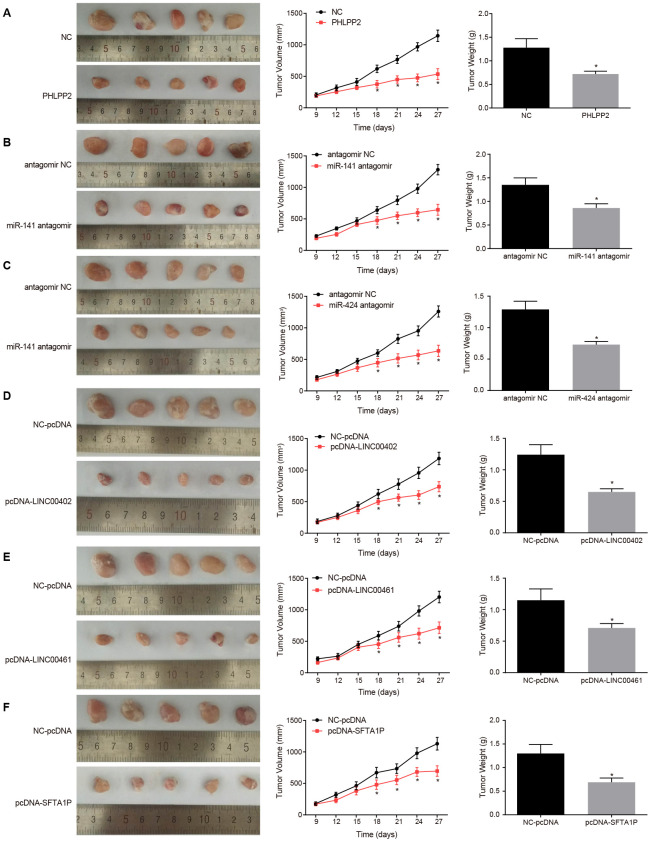
**Overexpression of PHLPP2, LINC00402, LINC00461, and SFTA1P or the inhibition of miR-141 and miR-424 inhibits the growth of formed tumor and reduces the tumor volume.** (**A**) Representative images, tumor volume, and tumor weight of formed tumors in nude mice harboring NC or PHLPP2. (**B**) Representative images, tumor volume, and tumor weight of formed tumors in nude mice harboring antagomir NC or miR-141 antagomir. (**C**) Representative images, tumor volume and tumor weight of formed tumors in nude mice harboring antagomir NC or miR-424 antagomir. (**D**) Representative images, tumor volume, and tumor weight of formed tumors in nude mice harboring NC-pcDNA or pcDNA-LINC00402. (**E**) Representative images, tumor volume, and tumor weight of formed tumors in nude mice harboring NC-pcDNA or pcDNA-LINC00461. (**F**) Representative images, tumor volume and tumor weight of formed tumors in nude mice harboring NC-pcDNA or pcDNA-SFTA1P. * *p* < 0.05 *vs.* the NC-pcDNA group. Measurement data in this Figure were expressed as the mean ± standard deviation, with comparisons between two groups conducted by *t*-test (n = 5); the experiment was repeated three times independently.

The nude mice were inoculated with PHLPP2 and lncRNAs (LINC00402, LINC00461, and SFTA1P) or miRNA antagomir (miR-141 antagomir and miR-424 antagomir) into the right tumor, nine days after tumor formation. The results also confirmed overexpression of LINC00402, LINC00461, SFTA1P, and PHLPP2 or the inhibition of miR-141 and miR-424, could suppress the tumor growth (all *p* < 0.05) (Figure 11).

**Figure 12:**
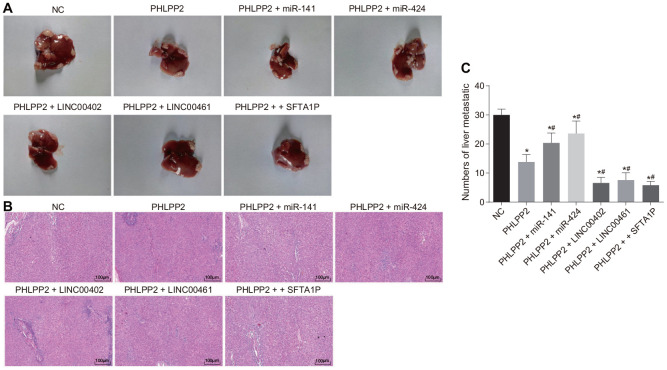
**The attenuation of PHLPP2 on tumor metastasis and formation is enhanced by the overexpression of LINC00402, LINC00461 and SFTA1P or reduced by miR-141 and miR-424.** Nude mice were injected with cell suspension carrying HT-29 cells transfected with NC, PHLPP2, PHLPP2 + miR-141, PHLPP2 + miR-424, PHLPP2 + LINC00402, PHLPP2 + LINC00461 or PHLPP2 + SFTA1P. (**A**) Representative images of the liver lesion in the liver tissues of nude mice. (**B**) HE staining of liver tissues (100 ×) in nude mice revealed the number of liver metastatic nodules. (**C**) The numbers of liver metastatic nodules in nude mice 8 weeks counted by HE staining. * *p* < 0.05 *vs.* the NC group; # *p* < 0.05 *vs.* the PHLPP2 group. Measurement data in this Figure were expressed as the mean ± standard deviation, and comparisons among multiple groups were conducted by One-Way ANOVA (n = 5); the experiment was repeated three times independently.

The lesions in the liver of nude mice were measured following the injection of lentiviral vector via the tail vein (Figure 12A–12C). The severity of liver lesion was relieved and the metastatic nodule was decreased in all groups (all *p* < 0.05). Compared with the PHLPP2 group, the severity of liver lesion was attenuated and the liver metastatic nodule was prominently decreased in the PHLPP2 + LINC00402, PHLPP2 + LINC00461 and PHLPP2 + SFTA1P groups (all *p* < 0.05), however, it was increased in the PHLPP2 + miR-141 and PHLPP2 + miR-424 groups (both *p* < 0.05). The statistical data of the tumor foci number were observed. HE staining also demonstrated that overexpression of LINC00402, LINC00461, and SFTA1P could enhance the inhibitory effect of PHLPP2 on tumor formation and metastasis.

### Overexpressed LINC00402, LINC00461, and SFTA1P upregulates PHLPP2 expression via miR-141 and miR-424

For further exploration regarding the effects of interaction among LINC00402, LINC00461, SFTA1P, miR-141, and miR-424 on PHLPP2 expression, subsequent experiments were performed. According to the above-described experiments, determining that overexpression of LINC00402, LINC00461, and SFTA1P could promote the transcription and translation of PHLPP2, the sh-LINC00402, sh-LINC00461, and sh-SFTA1P were delivered into cells. Our results showed that compared with the NC group, miR-141, and miR-424 expressions were significantly elevated in the sh-LINC00402, sh-LINC00461, and sh-SFTA1P groups (Supplementary Figure 4A) where PHLPP2 expression was significantly diminished (Supplementary Figure 4B, 4C). In the sh-LINC00402 + miR-141 inhibitor and sh-LINC00461 + miR-141 inhibitor groups, PHLPP2 was upregulated when compared with the sh-LINC00461 group. Similar uptrend of PHLPP2 expression was also observed in the sh-SFTA1P + miR-424 inhibitor than that in the sh-SFTA1P group, indicating that overexpressed LINC00402 and LINC00461 upregulated the PHLPP2 via miR-141 and miR-424. Furthermore, a reduction of PHLPP2 expression was found in the miR-141 and miR-424 groups yet no significant difference was witnessed regarding the expression of miR-141 and miR-424 in the PHLPP2 group (Supplementary Figure 4C, 4D), suggesting that miR-141 and miR-424 negatively regulated the PHLPP2 expression. Conclusively, these findings demonstrated that LINC00402, LINC00461, and SFTA1P mediated the PHLPP2 expression in a positive manner via miR-141 and miR-424.

## DISCUSSION

PHLPP has been identified to directly dephosphorylate the hydrophobic motif of Akt (Ser473 on Akt1), resulting in inhibition of kinase activity and promotion of apoptosis [22]. Although PHLPP isoforms, PHLPP1, and PHLPP2, selectively dephosphorylate the same site on Akt, it has been also reported to control different downstream substrates. PHLPP1 specifically modulates the phosphorylation of HDM2 and GSK-3a through Akt2 and plays an important role in glucose homeostasis, whereas PHLPP2 specifically modulates the phosphorylation of p27 through Akt3 and controls cell survival [23]. However, failure to terminate the Akt pathway results in increased cell growth, proliferation, and inhibition of apoptosis. Thus, the PHLPP2-Akt3-p27 signaling complex is considered to have a crucial role in tumorigenesis.

In consent with our present study, a tumor suppressor in colon cancer, PHLPP2 has been reported to undergo progressive depletion in colon cancer cells [9, 24]. Moreover, our study also identified PHLPP2 as a protective candidate gene in colon cancer by DEGs whilst our *in vivo* and *in vitro* results confirmed the tumor-suppressive role of PHLPP2 in colon cancer. It is well-known that miRNAs regulate the protein-coding genes by degrading mRNAs or inhibiting the translation of mRNAs on target RNA transcripts [25, 26]. While PHLPP2 has been identified as a target gene of various miRNAs [24, 27], playing a crucial role in cancer cell proliferation and apoptosis. Our analysis indicated that PHLPP2 might be the target of both miR-141 and miR-424, which have been reported to increase in colon cancer [14, 28]. The luciferase experiment confirmed that both miR-141 and miR-424 could bind to PHLPP2 mRNA and inhibited its expression. Moreover, our results from colon cancer also confirmed that expression of PHLPP2 was decreased in colon cancer, while the expression of miR-141 and miR-424 was increased. *In vivo* and *in vitro* results further verified the tumor-suppressive role of PHLPP2 by inhibiting proliferation, migration, invasion, and EMT in tumor formation. Hence, our result confirmed that both miR-141 and miR-424 could bind to PHLPP2 mRNA and inhibit the PHLPP2 tumor-suppressive effect in colon cancer. Notably, overexpression of PHLPP2, as well as the inhibition of miR-141 or miR-424 could inhibit the proliferation, migration, invasion, and EMT of colon cancer cells by inhibiting the activation of the Akt3-p27 pathway.

**Figure 13:**
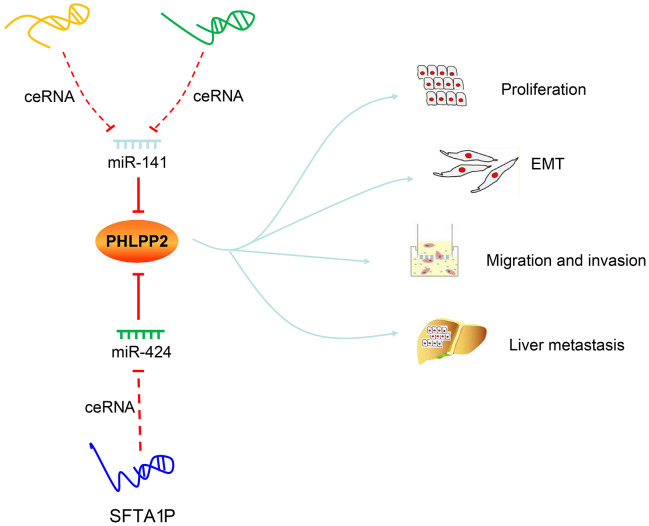
**Regulatory mechanisms of LINC00402, LINC00461, SFTA1P, miR-141, miR-424 and PHLPP2 in colon cancer progression and metastasis.** LINC00402 and LINC00461 competitively bind to miR-141, and SFTA1P competitively binds to miR-424 to regulate the transcription and translation of PHLPP2, thereby suppressing the proliferation, EMT process, migration, and invasion, preventing liver metastasis of colon cancer cells.

Importantly our study unravels the crucial role of LINC00402, LINC00461, and SFTA1P as ceRNAs, which further possess the ability to inhibit miR-141/miR-424 to suppress PHLPP2 or promote EMT by competitively binding to them. Moreover, it has been reported that SFTA1P could lead to the inhibition of cell migration and invasion in lung adenocarcinoma [29]. However, to date, numbers of lncRNAs have been reported to be involved in the tumorigenesis and progression of the tumor via the ceRNA mechanism [30–33]. The fact that several lncRNAs have been detected in the mediation of EMT, invasion, and metastasis in colon cancer [34–36], however, little is known about their definite activation in a tumor. Thus, the detailed mechanism of LINC00402, LINC00461, and SFTA1P remained unknown. Intriguingly, our results from both HT-26 cells and tumor xenograft mice

proved that the mechanism i.e., inhibition of PHLPP2 or regulation of EMT by miR-141/miR-424 were both suppressed by three lncRNAs. Considering the facts that both PHLPP2 and three lncRNAs are targets of miR-141/miR-424, we come to a consensus thatLINC00402, LINC00461, and SFTA1P possess the ability to act as ceRNAs and improve the expression of PHLPP2 through ceRNA mechanism by binding to miR-141 or miR-424, which ultimately lead to the suppression of pathogenesis in colon cancer (Figure 13).

## CONCLUSIONS

The ceRNA mechanism on tumorigenesis, cancer progression and cancer therapy is still under investigation. Our work attempted to bring attention to the crucial role of PHLPP2 in the regulation of ceRNA networks via regulating the miRNAs at transcriptional and post-transcriptional levels. Nevertheless, additional clinical studies are prerequisites to validate this identified network. Conclusively, through the combination of multiple RNAs regarding the initiation and progression of colon cancer, the present study provides a potential therapeutic target for colon cancer.

## MATERIALS AND METHODS

### Differentially expressed genes (DEGs) screening

The gene expression microarray datasets associated with colon cancer were collected from the gene expression omnibus (GEO) database (http://www.ncbi.nlm.nih.gov/geo). After standardization and pre-processing, DEGs were screened out using the limma package in R software [37] and the differential gene expression heatmap was drawn. The *p-*value < 0.05 and |log2 (fold change)| > 2.0 were set as the threshold. Venn diagrams (http://bioinformatics.psb.ugent.be/webtools/Venn/) were applied for the intersection analysis of the first 150 DEGs of 2 colon cancer microarray datasets (GSE41328 and GSE89076). Meanwhile, the gene expression data were downloaded from The Cancer Genome Atlas (TCGA, http://cancergenome.nih.gov/), Moreover, the differential analysis was conducted using the edgeR package of the R software [38]. False-positive discovery rate (FDR) correction was applied on a *p*-value basis in connection with the package multi-test. The DEGs were screened out with FDR < 0.05 and |log2 (fold change)| > 2.0 as the threshold.

### Enrichment analysis of Kyoto encyclopedia of genes and genomes (KEGG) pathways

Enrichment analysis of KEGG pathways was performed in relation to the DEGs on David’s website (https://david.ncifcrf.gov/summary.jsp).

### Prediction of miRNAs and lncRNAs

MiRNAs binding to DEGs were predicted on the miRDB website (http://www.mirdb.org/), miRTarBase website (http://mirtarbase.mbc.nctu.edu.tw/php/search.php) and TargetScan website (http://www.targetscan.org/vert_71/). Predictions regarding miRNAs binding to lncRNAs were conducted using the mircode website (http://www.mircode.org/).

### Study subjects

A total of 135 patients diagnosed with colon cancer at the general surgery department of Changzheng Hospital, the Second Military Medical University were randomly selected from March 2010 to October 2011, of which included 83 males and 52 females, aged between 30 and 81 years, with a mean age of 56.4 ± 7.2 years. Colon cancer tissues and adjacent tissues were surgically resected from all participating subjects. All patients had previously been pathologically diagnosed with colon cancer and did not receive any treatment for colon cancer within the last three months, and had complete medical records. Patients with metastatic colon cancer, patients with concurrent immune system diseases, endocrine system diseases, connective tissue diseases or anemia, patients with concurrent acute intestinal obstruction, intestinal perforation or intra-abdominal infection, patients who received radiotherapy, chemotherapy or both before surgery as well as patients lost to follow-up or with incomplete medical results were excluded from the study. The follow-up process was conducted for all patients, the duration of which ranged between 6 to 72 months. There were 10 patients lost to follow-up, with the follow-up rate determined to be 92.59%. The survival analysis was performed using a Kaplan-Meier curve. During the process of the entire follow-up period, the follow-up ended in the event of death or the final date of the last follow-up in March 2016. The overall survival of patients was defined as the duration from the initial date of surgery to the subsequent date of death.

### Immunohistochemistry assay

Immunohistochemistry assays were performed according to the published protocols [39]. In brief, both the colon cancer and adjacent tissue biopsy specimens were fixed in 4% neutral buffered formalin (DF0113, Solarbio, Beijing, China), embedded with paraffin and then sliced into serial sections (4 μm in thickness). The rabbit anti-human PHLPP2 antibody (1: 50, ab77665, Abcam, Inc., Cambridge, UK) was added for incubation at 4°C overnight, followed by 3 phosphate buffer saline (PBS) washes. The biotin-labeled goat anti-rabbit immunoglobulin G (IgG) (1: 1000, ab6721, Abcam) as a secondary antibody was added to the sections for an additional incubation process at 4°C for 40 min. The final results of the immunohistochemistry assay were scored independently by three pathologists for all clinical and biological data via a blind method. The staining intensity was graded based on a four-tier classification system: minus = 0, weakest = 1, medium = 2, strongest = 3. The sections scored 0 - 1 point was included in the low PHLPP2 group while those scored 2 - 3 points in the high PHLPP2 group [40, 41].

### In situ hybridization (ISH)

ISH assays were performed according to published protocols [42]. The tissues stained with BCIP/NBT were blue in color, while nuclear fast red staining was red in color. The results were scored by two independent pathologists. Positive cells were represented by blue in plasma. Five fields were randomly selected for each film under the microscope (200 ×), with the percentage of positive cells subsequently calculated. Negative was defined as positive cells < 5%, while positive was defined by positive cells ≥ 5%.

### Fluorescence in situ hybridization (FISH)

FISH assays were performed according to published protocols [43]. FISH technology was utilized for the subcellular localization of lncRNAs in the colon cancer cells, which was conducted according to the instructions of the Ribo^TM^ lncRNA FISH probe Mix (Red) (RiboBio, Guangzhou, China). Five fields were selected for each specimen under the fluorescence microscope (Olympus, Japan), observed, and photographed accordingly.

### Dual-luciferase reporter gene assay

To elucidate the binding relationship of lncRNAs to miRNAs as well as targeting the relationship of miRNAs to PHLPP2, the gene fragments of PHLPP2 containing the target site of miRNAs in the 3’untranslated region were artificially synthesized and then inserted into pMIR-reporter (Huayueyang Biotechnology Co., Ltd., Beijing, China). The luciferase activity was detected using the Dual-Luciferase Reporter Assay System (Promega; 48 h after transfection) according to the manufacturer’s instructions.

### Cell culture and transfection

Normal colon epithelial cell line CCD 841 CoN purchased from ATCC (American type culture collection, Manassas, VA, USA) was cultured in Dulbecco's modified eagle medium (DMEM) containing 10% fetal calf serum (FCS). Human colon cancer cell lines HT-29 and SW480 (both bought from ATCC) were cultured in Roswell Park Memorial Institute 1640 (11875119, GIBCO BRL, Grand Island, NY, USA) containing 10% FCS (10099141, GIBCO BRL). Human colon cancer cell line SW620 (ATCC) were cultured in L15 (11415-064, GIBCO BRL) containing 10% FCS. Human colon cancer cell line Lovo (ATCC) was cultured in F12K medium (21127022, GIBCO BRL) containing 10% FCS. Human colon cancer cell line HCT-116 (ATCC) was cultured in DMEM/F12 (11330057, GIBCO BRL) containing 10% FCS. Three to four days later, the cells were sub-cultured in a 37°C, 5% CO_2_ incubator (thromo3111, Jinan Beisheng Medical Equipment Co., Ltd., Shandong, China), with the renewal of medium at regular 2 days intervals. The cells were transiently transfected with the corresponding vector using the Lipofectamine 3000 Transfection Reagent (Invitrogen, Carlsbad, CA, USA) according to the manufacturer’s instructions. The transfection was assigned into 8 sets as follow: (1) negative control (NC), PHLPP2, PHLPP + miR-141 and PHLPP + miR-424; (2) NC-pcDNA, PHLPP2, NC-inh (NC for miR-141 inhibitor), miR-141 inhibitor, miR-424 inhibitor, PHLPP2 + miR-141, and PHLPP2 + miR-424; (3) NC, si-PHLPP2 (si-PHLPP2: 5’-CGAAUCCUACUGUCUGGCAUCUAUA-3’); (4) NC, PHLPP2, PHLPP2 + LINC00402, PHLPP2 + LINC00461, and PHLPP2 + SFTA1P; (5) NC-pcDNA, pcDNA-LINC00402, NC-sh, sh-LINC00402, and pcDNA-LINC00402 + miR-141; (6) NC-pcDNA, pcDNA-LINC00461, NC-sh, sh-LINC00461, and pcDNA-LINC00461 + miR-141; (7) NC-pcDNA, pcDNA-SFTA1P, NC-sh, sh-SFTA1P, and pcDNA-SFTA1P + miR-424; (8) NC, PHLPP2, PHLPP2 + pcDNA-LINC00402, PHLPP2 + pcDNA-LINC00461, and PHLPP2 + pcDNA-SFTA1P, respectively.

### Reverse transcription quantitative polymerase chain reaction (RT-qPCR)

The RT-qPCR assays were performed according to published protocols [44, 45]. The total RNA of the cells was extracted using a Trizol kit (16096020, Thermo Fisher Scientific, Waltham, MA, USA). The cDNA Reverse Transcription kit (Applied Biosystems) was applied for cDNA synthesis with 5 μg of RNA, while 25 μL of PCR amplification system for the target gene was comprised of 300 ng cDNA. Primers are listed in Table 2. The relative expression of each target gene was calculated by the 2^-ΔΔCt^ with U6 serving as an internal reference for miRNA and glyceraldehyde-3-phosphate dehydrogenase (GAPDH) for other genes.

**Table 2:** The enrichment analysis of lyoto Encyclopedia of genes and genomes pathways on david website.

**Pathway**	***P* value**	**Gene**
Nitrogen metabolism	0.010983582	*CA7*
Hippo signaling pathway	0.017402099	*AJUBA*
Pathogenic Escherichia coli infection	0.032046102	*CLDN1*
Leukocyte transendothelial migration	0.020092127	*CLDN1*
Hepatitis C	0.026220505	*CLDN1*
Cell adhesion molecules	0.0317101	*CLDN1*
Tight junction	0.042318542	*CLDN1*
Hippo signaling pathway	0.049596277	*AJUBA*
PI3K-Akt signaling pathway	0.02365356	*PHLPP2*

Note: PI3K, Phosphatidylinositol-3-kinase; CA7, Carbonic anhydrase 7; CLDN1, Claudin-1; PHPPL2, pleckstrin homology domain leucine-rich repeat protein phosphatase.

### Western blot analysis

Western blot assay was performed according to published protocols [46, 47]. The total protein was extracted from cells and tissues by Radio Immunoprecipitation Assay lysis buffer (R0010, Solarbio) containing phenylmethylsulfonyl fluoride. After electrophoresis, proteins were transferred to nitrocellulose membranes. Membranes were incubated in Western Blocking Reagent (Roche Applied Science) and treated with antibodies (all purchased from Abcam Inc., Cambridge, MA, USA). Primary antibodies against E-cadherin (ab76055, 1: 500), N-cadherin (ab18203, 1: 1000), Vimentin (ab8978, 1: 1000), B-cell lymphoma-2 associated protein X (Bax) (ab77566, 1: 500), cleaved Caspase-3 (ab32042, 1:500), PHLPP2 (ab71973, 1: 2000), phosphorylated-Akt3 (p-Akt3)(1: 500, ab192623), p-Akt1 (1: 500, ab81283), p27(1: 500, ab75908), and GAPDH (ab9485, 1: 2000) were used. Horseradish peroxidase-conjugated secondary antibodies against rat, goat or rat IgG were added (Dako, Life technologies). After antibody treatment, blots were developed using an enhanced chemiluminescence Western Blotting Detection System (GE Healthcare). The Bio-Rad image analysis system (Bio-Rad, Hercules, CA, USA) was utilized for the photograph and Quantity One v4.6.2 software was applied for protein band analysis. The ratio of gray values of the protein band to be tested in relation to the GAPDH protein band was considered to be the relative protein expression.

### Immunofluorescence assay

After 48 hours of transfection, the cells fixed in 4% paraformaldehyde for 30 minutes and then treated with 0.2% TritonX-100 at room temperature for 15 minutes and treated with 3% bovine serum albumin at 4°C for 30 minutes. The primary antibodies (all purchased from Abcam Inc., Cambridge, UK) E-cadherin (ab76055, 1: 200), N-cadherin (ab18203, 1: 200) and Vimentin (ab8978, 1: 500) were added for incubation at 4°C overnight. Subsequently, fluorescence secondary antibody (1: 500) was added for 2-hours incubation at room temperature under dark conditions. Then, 4',6-diamidino-2-phenylindole (ab104139, 1: 100, Abcam) was added to the cells for a 10-minutes reaction at room temperature under dark conditions. Lastly, the cells were mounted with neutral gum after 3 PBS rinses (5 min per time). An inverted fluorescence microscope was applied for observation.

### 5-ethynyl-2’-deoxyuridine (EdU) labeling assay

EdU staining protocol was performed following the manufacturer’s instructions (Click-iTEdU, Invitrogen). The images obtained with red light (550 nm) were applied to reveal the cells stained in red were proliferative cells, while the images obtained with purple light (350 nm) indicated that cells with purple staining were total cells. The number of cells stained with EdU (proliferative cells) and that stained with Hoechst (total cells) was counted separately. The proliferation rate = proliferative cells/total cells × 100%.

### Scratch test

Scratch test assays were performed according to the published protocols [48, 49]. At the time point of the 0^th^ hours and 48^th^ hours, images of the cells were obtained and the migration distance of cells was measured using the Image-Pro Plus Analysis Software (Media Cybernetics, Silver Springs, MD, USA).

### Transwell assay

Transwell assays were performed according to published protocols [50]. Five fields were randomly selected under an inverted microscope (XDS-800D, Shanghai Caikon Optical Instrument Co., Ltd., Shanghai, China). The number of cells passing through Matrigel in each group was counted and regarded as an index in the evaluation process of their invasion ability. The wells were preset in triplicate for each group followed by the calculation of the average value.

### Flow cytometry

After 48 hours of cell transfection, Annexin V-FITC/PI double staining was conducted for the detection of cell apoptosis. Cell apoptosis was detected at an excitation wavelength of 488 nm by a flow cytometer (Becton, Dickinson and Company, NJ, USA).

### RNA immunoprecipitation (RIP) assay

RIP assays were performed according to published protocols [51]. The binding of lncRNAs to Argonaute 2 (Ago2) was identified using a RIP reagent kit (Merck Millipore, Billerica, MA, USA). The Ago2 antibody (ab32381, 1: 50, Abcam) in this assay was added, IgG (ab109489, 1: 100, Abcam) was used as NC.

### RNA pull-down

RNA pull-down assays were performed according to published protocols [52]. The RNA pull-down was conducted using a Magnetic RNA-Protein Pull-Down Kit (Pierce, Rockford, IL, USA).

### Tumor xenograft in nude mice

Totally, 60 specific pathogen-free (SPF) male nude mice (weighing 14-16 g, aged 5 weeks) were purchased from Shanghai SLAC Laboratory Animal Co., Ltd. (Institute of Biochemistry and Cell Biology, Shanghai Institutes for Biological Sciences, Chinese Academy of Sciences, Shanghai, China) raised under constant controlled temperature (25-27°C) and humidity (45 - 50%) were included for the purposes of the study. Tumor xenograft assays were performed according to published protocols [53]. After anesthesia, cells at the logarithmic growth phase following transfection were re-suspended in 50% Matrigel (BD Biosciences, Bedford, MA, USA) with the cell concentration adjusted to 2 × 10^6^ cells/mL. Then, 0.2 mL single-cell suspension (containing 4 × 10^5^ cells) was subcutaneously inoculated into the 60 nude mice at the left sub-axilla. On the 9^th^, 12^nd^, 15^th^, 18^th^, 21^st^, 24^th^, and 27^th^ day of inoculation, the width (W) and length (L) of the tumor were measured using a digital vernier caliper followed by determination of the tumor volume (V). Formula: V (mm^3^) = (L × W^2^)/2. The experimental grouping included 2 sets (n = 5): (1) NC-pcDNA, PHLPP2, NC-inh, miR-141 inhibitor, miR-424 inhibitor, PHLPP2 + miR-141, and PHLPP2 + miR-424; (2) NC, pcDNA-LINC00402, pcDNA-LINC00461, pcDNA-SFTA1P, and pcDNA-LINC00402 + pcDNA-LINC00461 + pcDNA-SFTA1P.

Another 30 SPF nude mice were subjected to subcutaneous inoculation of 0.2 mL cell suspension 0.2 mL of single-cell suspension (2 × 10^6^ cells/mL) at the left sub-axilla and right sub-axilla, respectively. About 9 days later, after confirmation of tumor formation, the nude mice were inoculated with 10 μg of PHLPP2 and lncRNAs (LINC00402, LINC00461, and SFTA1P) or with 1.5 nmol miRNA antagomir (miR-141 antagomir and miR-424 antagomir) into the right tumor. Afterward, the nude mice were inoculated with NC plasmid into the left location as control. The inoculation process was performed 5 times after the initial measurement of the width and length every three days. The experimental grouping included 6 pairs (n = 5): (1) NC and PHLPP2; (2) antagomir NC and miR-141 antagomir; (3) antagomir NC and miR-424 antagomir; (4) NC-pcDNA and pcDNA-LINC00402; (5) NC-pcDNA and pcDNA-LINC00461 (6) NC-pcDNA and pcDNA-SFTA1P.

Then, thirty-five 5-week SPF male nude mice were intravenously inoculated with 0.1 mL of single-cell suspension (1 × 10^6^ cells/mL) from each group after a 48-hours period of transfection at the tail vein. In the 8^th^ week of inoculation, liver tissues of nude mice were collected and sliced into sections. Hematoxylin-eosin (HE) staining was subsequently utilized to tally the number of metastatic tumor nodes in the liver. Seven experimental groups were set (n = 5), including the NC, PHLPP2, PHLPP2 + miR-141, PHLPP2 + miR-424, PHLPP2 + LINC00402, PHLPP2 + LINC00461, and PHLPP2 + SFTA1P groups.

### Statistical analysis

All experimental data were processed by the SPSS 21.0 statistical software (IBM Corp. Armonk, NY, USA). Measurement data were expressed as the mean ± standard deviation, while normal distribution was assessed using the D'Agostino and Pearson omnibus normality test. Comparisons of normally distributed measurement data between the two groups were assessed by paired *t*-test; comparisons of those among multiple groups were conducted using One-Way Analysis of Variance (ANOVA); while the pairwise comparison of the mean values among the experimental groups was performed using the Tukey post-hoc test. With regard to the data that did not follow a normal distribution, Dunn's multiple comparison post-hoc test in the Kruskal-Wallis test was applied for comparisons of survival, while Pearson's Correlation Analysis was conducted to analyze the correlation between PHLPP2 and miRNAs. A comparison of survival time among patients with high and low expressions of PHLPP2 was performed by Kaplan-Meier survival analysis. *p* < 0.05 was indicative of statistical significance.

### Ethics statement

The present study was conducted under the approval of the Ethics Committee of Changzheng Hospital, the Second Military Medical University. All participating patients provided and subsequently signed written informed consent documentation. All experimental animal procedures were performed in strict adherence to *Guide for the Care and Use of Laboratory Animals* by US National Research Council (Washington, DC: The National Academies Press, 2011), with the conducted experiments performed in accordance with the three Rs principles (reduction, replacement, and refinement).

## Supplementary Material

Supplementary Figures
