# Supplementary material for: PHLPP2 is regulated by competing endogenous RNA network in pathogenesis of colon cancer
Source: Aging (Albany NY). 2020 Jul 7;12(13):12812–40. doi: 10.18632/aging.103246 (PMC7377866; doi:10.18632/aging.103246)
Supplement: Supplementary Figures [file aging-12-103246-s001..pdf]

SUPPLEMENTARY FIGURES

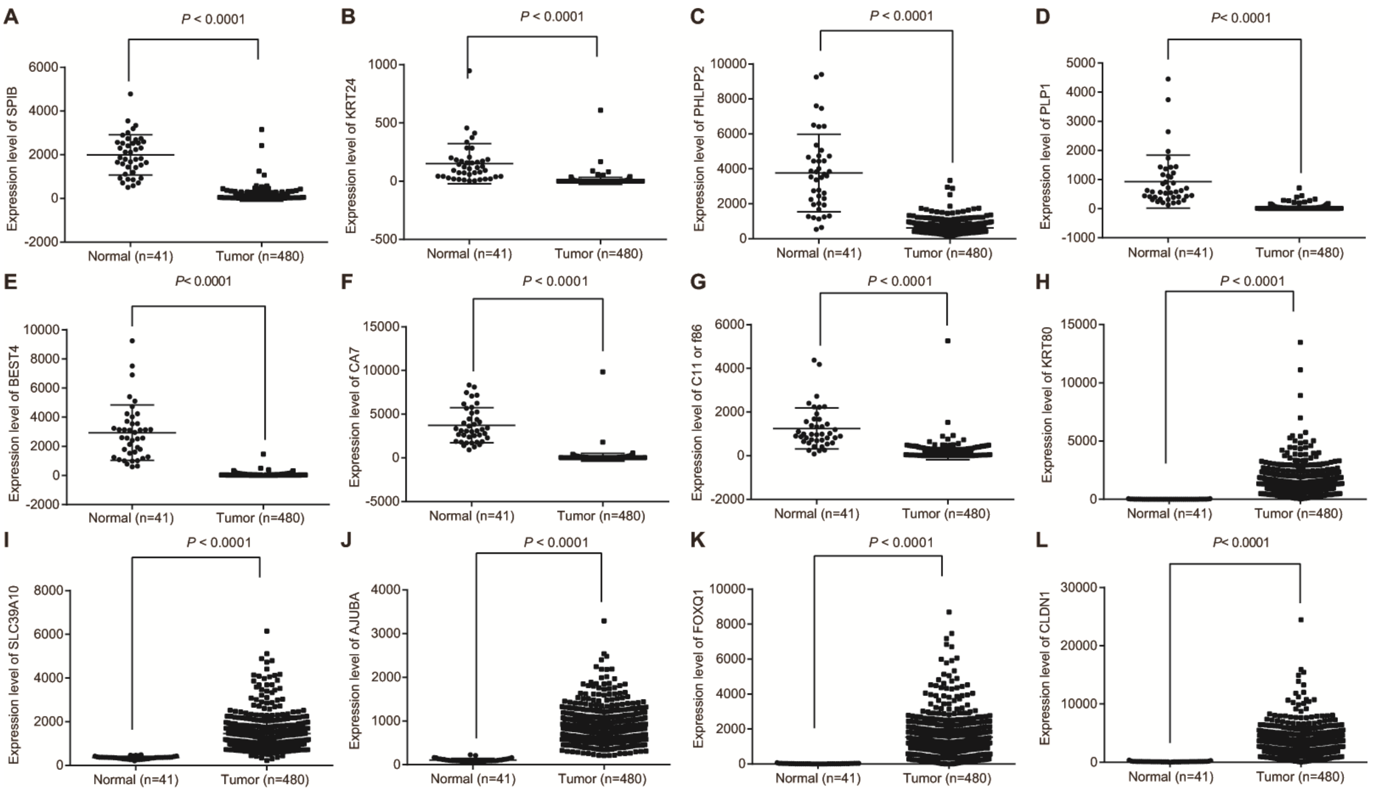

Supplementary Figure 1. Expression of DEGs in the colon cancer and adjacent tissues from the TCGA database. (**A**) SPIB expression. (**B**) KRT24 expression. (**C**) PHLPP2 expression. (**D**) PLP1 expression. (**E**) BEST4 expression. (**F**) CA7 expression. (**G**) C11orf86 expression. (**H**) KRT80 expression. (**I**) SLC39A10 expression. (**J**) AJUBA expression. (**K**) FOXQ1 expression. (**L**) CLDN1 expression.

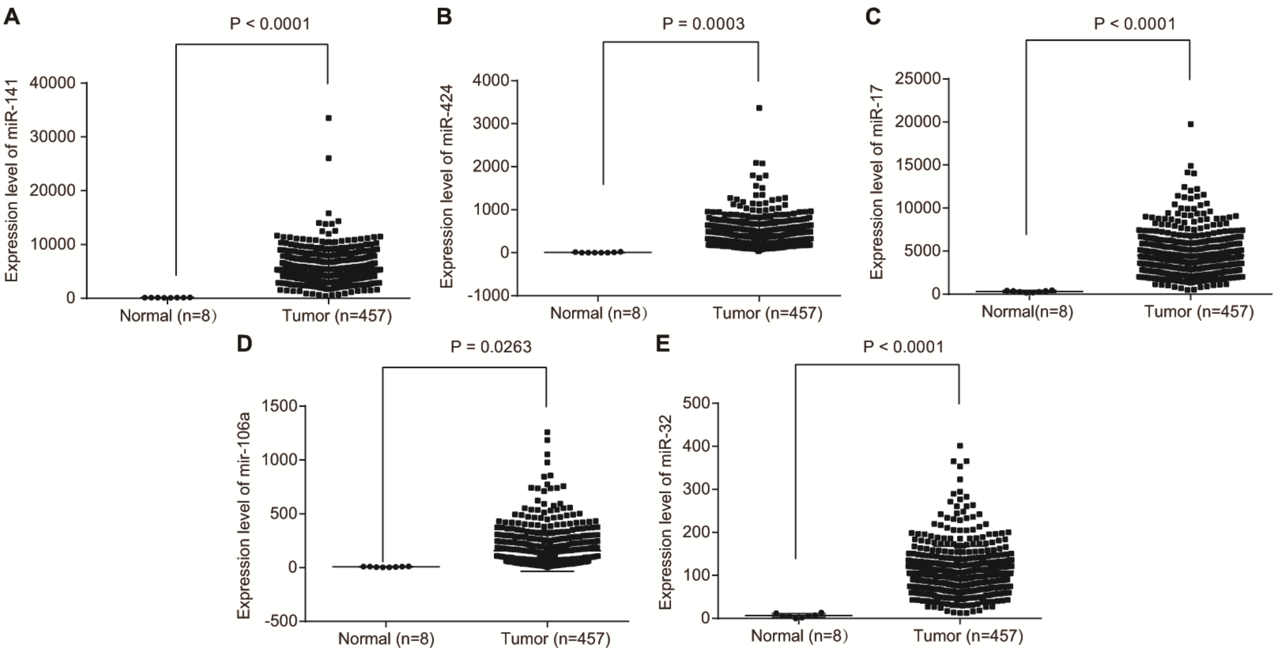

Supplementary Figure 2. Expression of miRNAs in colon cancer and adjacent tissues from the TCGA database. (**A**) miR-141 expression. (**B**) miR-424 expression. (**C**) miR-17 expression. (**D**) mir-106a expression. (**E**) miR-32 expression.

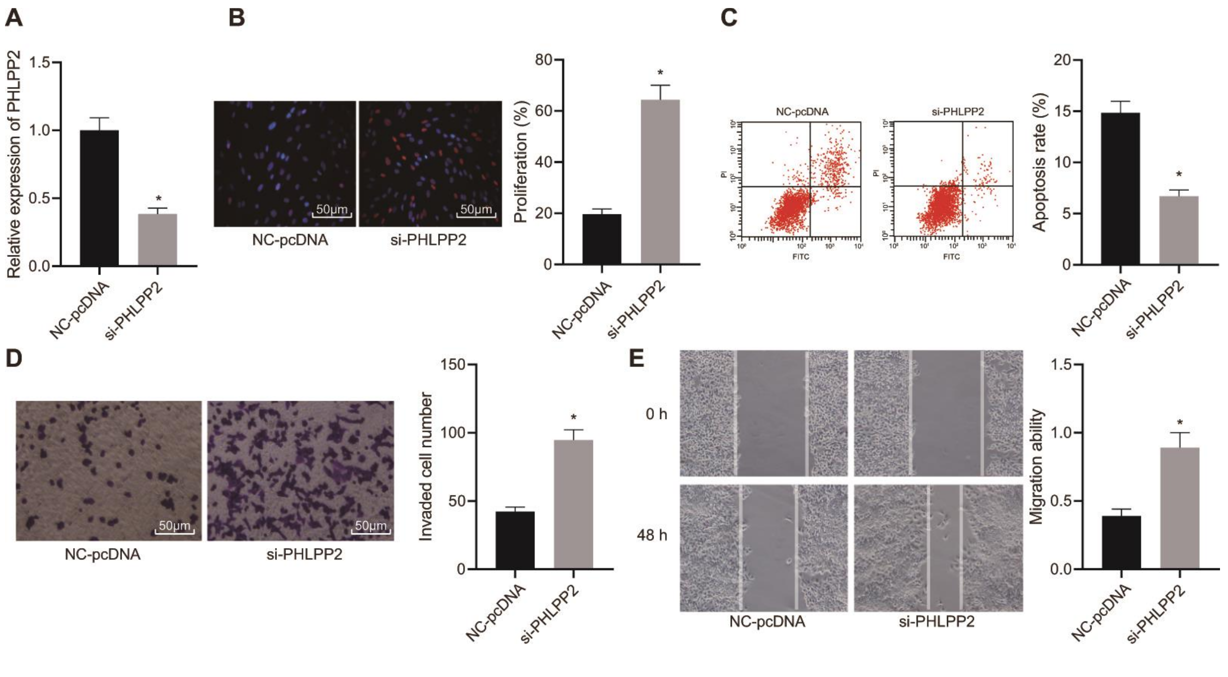

Supplementary Figure 3. Overexpression of PHLPP2 inhibits SW620 cell proliferation, migration, and invasion while promoting cell apoptosis. (**A**) Transfection efficiency of PHLPP2 detected by RT-qPCR. (**B**) SW620 cell migration detected by EdU assay. (**C**) SW620 cell apoptosis detected by flow cytometry. (**D**) SW620 cell invasion detected by Transwell assay. (**E**) SW620 cell migration detected by scratch test. * $p < 0.05$ *vs.* the NC-pcDNA group. Measurement data in this Figure were expressed as the mean ± standard deviation, and comparisons among multiple groups were conducted by One-Way ANOVA. The experiment was repeated three times independently.

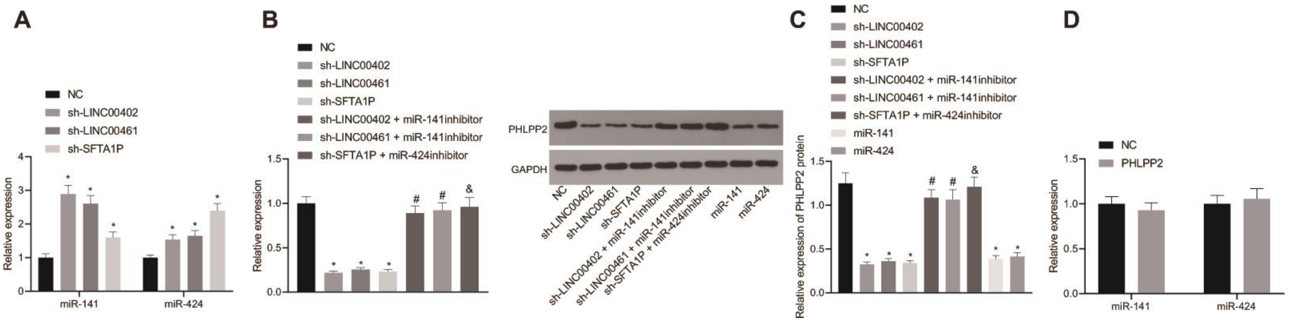

Supplementary Figure 4. miR-141 and miR-424 are required for the regulatory role of LINC00402, LINC00461, and SFTA1P on PHLPP2 expression in colon cancer. (**A**) miR-141 and miR-424 expression determined by RT-qPCR. (**B**) PHLPP2 mRNA expression determined by RT-qPCR. (**C**) PHLPP2 protein level normalized to GAPDH determined by Western blot analysis. (**D**) miR-141 and miR-424 expression determined by RT-qPCR. * $p < 0.05$ *vs.* the NC group. # $p < 0.05$ *vs.* the sh-LINC00402 and sh-LINC00461 groups. & $p < 0.05$ *vs.* the sh-SFTA1P group. Measurement data in this Figure were expressed as the mean ± standard deviation, and comparisons among multiple groups were conducted by One-Way ANOVA. The experiment was repeated three times independently.
